# Critical dimensions in strengthening education and instructor training using fuzzy based decision algorithm and CRITIC WASPAS method

**DOI:** 10.1038/s41598-025-12393-w

**Published:** 2025-08-06

**Authors:** Hui Zhou, RenGuo Yang

**Affiliations:** 1 Department of Endocrinology, Sichuan Provincial People’s Hospital, School of Medicine, University of Electronic Science and Technology of China, Chengdu, 610072 Sichuan China; 2 Department of Infectious Disease, Sichuan Provincial People’s Hospital, School of Medicine, University of Electronic Science and Technology of China, Chengdu, 610072 China

**Keywords:** Circular pythagorean fuzzy information, Frank aggregation operators, CRITIC-WASPAS method and decision support system, Engineering, Mathematics and computing

## Abstract

Quality education and instructor training are foundational to the development of any society, as they directly influence the effectiveness of learning and the overall performance of educational systems. Decision-making is crucial in enhancing quality education and instructor training by ensuring teaching policies. This article modifies some robust mathematical methodologies of the fuzzy framework and decision-making techniques to aggregate an authentic ranking of preferences. We also explore a novel approach to the circular pythagorean fuzzy set (Cr-PyFS) that is used to manage uncertainty and vagueness in complicated real-life applications. Some flexible operations of Frank t-norm and t-conorm are also formulated under the system of circular pythagorean fuzzy (Cr-PyF) information. Furthermore, we derive a list of mathematical aggregation operators such as circular pythagorean fuzzy frank weighted average (Cr-PyFFWA) and circular pythagorean fuzzy frank weighted geometric (Cr- PyFFWG) operators with prominent properties. Additionally, a decision-making approach to the criteria importance through the intercriteria correlation (CRITIC) method is adopted to investigate the weight of criteria by incorporating the Cr-PyF situations. Moreover, an optimization technique of the weighted aggregated sum product assessment (WASPAS) method is established to rank alternatives under different conflicting criteria. A numerical example is constructed to examine suitable training institutes to improve instructor skills and expertise. The comparative study is also established to demonstrate the superiority and validation of pioneering approaches with existing terminologies. Finally, a summary of the article is presented at the end of the manuscript.

## Introduction

The quality of teacher training is crucial in higher education since it directly affects teachers’ capacity to engage with challenging material and encourage students to think more critically. Teachers need to be well-versed in current pedagogical techniques, possess content knowledge, and be able to support student-centred learning as curricula change to incorporate transdisciplinary knowledge, digital technology, and research-based learning. In addition to being knowledgeable about the subject matter, teachers who receive top-notch training are also adept at using cutting-edge teaching resources, accurately evaluating student performance, and modifying their lessons to accommodate different learning preferences. In higher education, when instructors’ responsibilities go beyond teaching to include mentoring, research advice, and curriculum building, this becomes even more crucial.

Additionally, long-term student performance, institutional reputation, and academic brilliance are all enhanced by high-quality teacher preparation in higher education. Schools that support their staff members’ ongoing professional development get more driven and self-assured educators, which enhances learning environments and student performance. Good training facilitates the integration of international educational trends, promotes the adoption of evidence-based teaching strategies, and cultivates reflective practice. Students benefit from a deeper, more applicable educational experience that equips them for both academic and professional problems. Therefore, improving the calibre of teacher preparation is a strategic necessity for furthering the objectives of contemporary educational systems.

The absence of ongoing and in-service training for employees results in diminished efficiency and effectiveness and a decline in their occupational identity. Taking into account that the standard of education in a community determines its advancement in all fields, offering in-service training for one of the most important requirements of the educational system is the presence of educators and school teachers. When talking about the importance of human resources, the emphasis mostly shifts to educated and well-cultivated individuals. This is due to the fact that these individuals are adequately qualified and skilled to contribute to constructive advancements in the many spheres of society, and often, training and investments in the human resources sector can help them acquire these abilities and skills^1^.

Most professionals in educational planning and management concur that using staff in-service training as an efficient mechanism is essential to striking a balance between organizational implementation processes and external development and initiatives^2^. In-service training, which is defined as training that takes place during working years, aims to increase job possibilities, raise knowledge of duties and obligations, develop skills, enable advancement to higher positions, and help people adjust to changing circumstances^3^.

The MADM approach’s primary goal is to use the decision-makers defined preferred values to identify the optimal option. Decision-makers struggle to analyze the finer points of each desire in real-world scenarios. Zadeh^4^ developed a new fuzzy set (FS) theory to address the previously mentioned problem by building upon conventional set theory. To handle complex and ambiguous data, many mathematicians employ the structure of fuzzy sets (FS), which incorporates numerous attributes within the framework of multiple fuzzy domains^5–7^.

Later, many researchers attempted to tackle various problems related to various domains, including artificial intelligence, machine learning, game theory, the medical profession, pattern recognition, and environmental sciences. One of the main implications of FS is the application of truth values, which are represented as [0,1], to human cognitive processes. Atanassov^8^ developed the concept of intuitionistic fuzzy set (IFS), adding non-membership grade in fuzzy set theory. Atanassov^9^ also provided some fundamental operational laws of union and intersection operations. Nonetheless, a wide range of applications were addressed by several mathematicians using the FSs and IFSs. Using rough fuzzy theory, Ahmmad et al.^10^ modified different mathematical approaches to cope with unpredictable and uncertain information from different human perspectives. Likewise, Rukhsar et al.^11^ initiated different power aggregation operators to expand the theoretical concepts of IFS to Circular-IFS (Cr-IFS). To deal with the uncertainty of human perspectives, the IFS has been extended to interval-valued IFS (IVIFS) by Atanassov^12^, linguistic interval-valued IFS^13^, q-rung orthopair fuzzy set (q-ROPFS) was developed by Yager^14^, and pythagorean fuzzy set (PyFS) was also introduced by Yager^15^. Hussain et al.^16^ utilized properties of Aczel Alsina t-norm and t-conorm under the system of PyFSs and complex PyFSs^17^ to investigate the ranking of preferences under different conflicting criteria.

In 2020, Atanassov^18^ introduced a novel theory of Cr-IFS to enhance the applicability of an IFS. A Cr-IFS contains an additional component like radius $r$ among the pair of membership and non-membership grades. The Cr-IFS can be used in many different areas of MCDM approaches, such as IFSs. Furthermore, research is being done on health tourism and the selection of medical waste disposal sites. Xu and Wen^19^ applied theoretical concepts of Cr-IFSs to assess different business proposals in terms of industrial symbiosis. A unique circular intuitionistic fuzzy MCDM technique was proposed by Çakır et al.^20^. Otay and Kahraman^21^ applied theoretical concepts of Cr-IFSs with the AHP and VIKOR approaches to solve a supplier selection problem. Alkan and Kahraman^22^ put forward the theory of the TOPSIS method to manage uncertainty and imprecision information under consideration of Cr-IFS. Özlü^23^ initiated the theory of the vector similarity measures, taking into account a picture fuzzy framework with hesitant models. Özlü^24^ modified Dombi mathematical models to investigate the ranking of alternatives using a group of expert’s opinions. Özlü^25^ proposed Aczel Alsina operators to accumulate information from different criteria and decision-making models. Rukhsar et al.^26^ elaborated concepts of Cr-IFS using the Frank aggregation operator for the selection of green suppliers with a decision support system. Numerous decision analytic procedures have been examined in various fields under different implications of fuzzy frameworks^27–29^.

The decision-makers need to aggregate the collected data using the aggregation operators (AOs), a vital tool for merging data in a single evaluation. According to the operators, decision-makers collect data for analysis using weighted averages, weighted sums, and geometric means. The AO is also essential for ranking and comparing complex data and making it understandable. Additionally, by providing the decision-making layout, the operators help stakeholders and businesses make the right choices. Consequently, the AOs are an essential tool for data merging and enhancing the efficacy of the decision-analysis process. Mathematicians developed a variety of methods to accomplish this by utilizing a number of fuzzy set strategies. Xu^30^ developed some well-known techniques using intuitionistic fuzzy information. Hussain et al.^31^ enhanced the applicability of Aczel Alsina operators under consideration of interval valued pythagorean fuzzy situations. The Bonferroni mean operators are used by Ali et al.^32^ to determine the optimal response. In order to build specific solutions, Wang and Wang^33^ looks into 2-tuple fuzzy linguistic sets. The Fermatean fuzzy theory was applied to derive advanced mathematical aggregation operators in^34^. A family of powerful Aczel Alsina operators was developed in^35^. A real-life application related to recycling waste materials was discussed in^36^. A potent approach of t-spherical fuzzy sets was combined with the theoretical concept of Dombi Hamy mean aggregation operators in^37^. Moreover, Hussain et al.^38^ developed the family of Schweizer-Sklar aggregation operators. In the context of complex fuzzy theory, Mahmood and Ur Rehman^39^ initiated different similarity measures to integrate uncertainty in human opinions. Özlü^40^ expanded the theoretical concepts of decision-making models by generalizing Dice measures with single-valued neutrosophic situations. ÖZLÜ and AKTAŞ^41^ defined correlation among different conflicting criteria under consideration of MCDM models. Karaaslan and Özlü^42^ discussed a real-life application related to clustering analysis and a decision support system. ÖZLÜ^43^ elaborated on different properties of Aczel Alsina operations to derive new mathematical models.

In 1995, Diakoulaki et al.^44^ established an efficient approach of the CRITIC method to determine weights for different factors in the context of a multi-criteria decision-making (MCDM) problem. This method integrates different results based on comparative ratio analysis and uses pairwise comparisons to ascertain the relative importance of every criterion. Kaur et al.^45^ combined theoretical concepts of two different approaches of the CRITIC method and the technique for order preference by similarity to ideal solution (TOPSIS) method for resolving the MCDM problem. Sleem et al.^46^ expanded a novel approach to the MADM problem to determine flexible ranking factors under different criteria. Mishra et al.^47^ employed an intelligent approach of the Fermatean fuzzy theory to resolve a hybrid decision-making model using the CRITIC method. Cui et al.^48^ also enhanced the capability of the decision-making problem. Das^49^ analyzed the surface water quality in the Mahanadi River based on the MCDM methodologies. A synopsis of MCDM techniques for managing logistics equipment selection was developed by Jusufbašić^50^. Ertemel et al.^51^ assessed different parameters of smartphone addiction using the pythagorean fuzzy-based CRITIC-TOPSIS method. Ranjan et al.^52^ integrated material selection using the decision support system of the CRITIC-MARCOS method. The theory of entropy measure was combined with the CRITIC method to find out the unknown degree of weight criteria in^53^. A hybrid decision-making model of the CoCoSo method combined with the CRITIC technique to determine a suitable ranking of preferences in^54^. Zafar et al.^55^ evaluated the performance of blockchain enterprises using the entropy-CRITIC method under the system of the MCDM problem.

Bączkiewicz et al.^56^ examined the systematic aspects of MCDM in an e-commerce recommendation system. Another novel decision-making approach to the MCDM problem was integrated by Kumar and Singh^57^. Saxena et al.^58^ designed a hybrid approach of the CRITIC-TOPSIS method for choosing the most effective growth based on software reliability and efficiency. A friendly green supplier selection was conducted by Vadivel et al.^59^. Furthermore, a novel theory of the WASPAS method was developed by Zavadskas and Turskis^60^. This approach offers a framework for evaluating and prioritizing options according to a number of factors. Decision science has greatly benefited from Zavadskas^61^ contributions to the creation of the WASPAS technique, which offers a methodical and weighted method to help DMs analyze complicated situations and make informed decisions. The WASPAS technique has been studied and used in a number of fields by researchers and practitioners, proving its versatility and value in solving practical decision-making issues.

### Research gap and motivation behind the proposed models

Despite significant advancements in intuitionistic and pythagorean fuzzy frameworks, they often fall short when dealing with highly ambiguous and circular data structures. The Cr-PyFS address this limitation by integrating circular representation with pythagorean conditions, allowing for a more flexible and realistic expression of expert uncertainty, hesitation, and opposition. However, there is still a gap in the literature regarding the application of Cr-PyFS in integrated decision models and their empirical validation across various domains. This motivates researchers to explore how Cr-PyFS can enhance the modelling of human judgment in complex environments, especially where interrelationships among criteria are nonlinear and uncertain.

On the other hand, while many aggregation operators have been proposed, Frank Weighted Average and Frank Weighted Geometric operators provide a more generalized and parameterized way to combine fuzzy data. Existing studies often overlook the flexibility and adaptability offered by Frank t-norms in capturing the decision-maker’s behavioral preferences. Moreover, the hybrid CRITIC method is used to determine weights of criteria and offers balanced evaluation through the WASPAS method, the decision-making process becomes more robust and efficient. There exists a research gap in applying Frank integrated mathematical approaches to resolve complicated real-life applications with more authenticity. The main focus and advantages of the article are discussed as follows:

- How to integrate human opinions without loss of information?
- How to develop new mathematical models using algebraic operations?
- How to aggregate expert’s opinions without the weight of criteria?
- How to compute the degree of unknown weights?
- Aggregation and computation process of real-life applications using advanced decision-making methodologies.

### Novelty and contributions

**Fig. 1:**
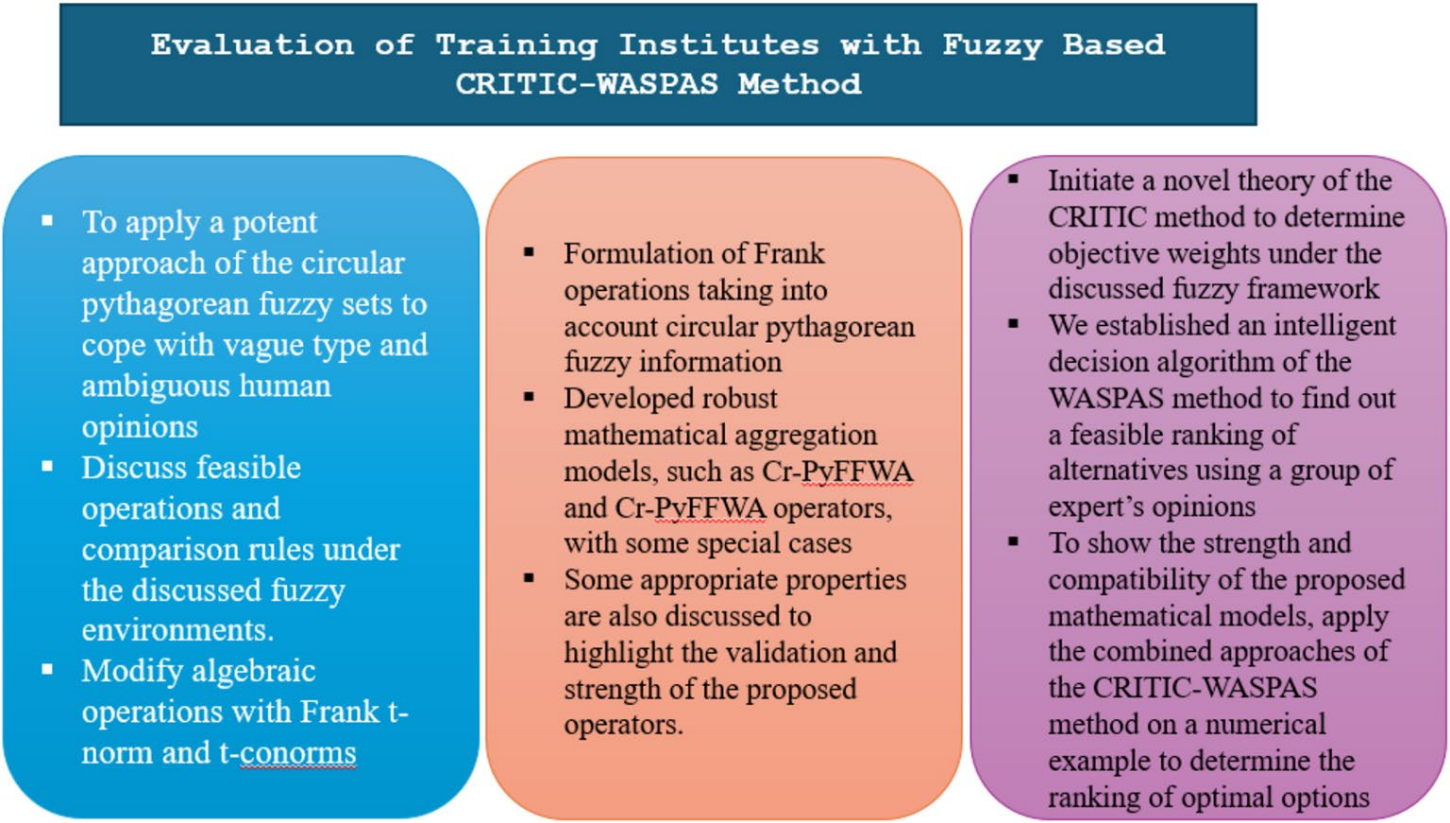
Explores primary contributions of the manuscript.

The novel multi-attribute decision-making (MADM) approach is proposed in this study as a remedy for the previously noted issue. The teacher’s training for quality education problems, which includes many alternatives and preferences, is tackled using the MADM problem. The wide range of applications in various fields in the literature shows the importance of fuzzy sets and aggregation operators. We have extended this work in the context of fuzzy sets by proposing new methods. This article represents the work on the Cr-PyFS using the Frank Aggregation operators and the CRITIC-WASPAS method. The notion that an efficient mathematical model, Cr-PyFS, can handle ambiguous and uncertain data involving human opinion is what drives this effort. In the decision analysis system, the Frank Aggregation offers a smoother approximation during the aggregation process and is more adaptable. The main features and contributions of this article are summed up as follows:

- To demonstrate the ambiguity and unpredictability of human opinion using the Cr-PyFS.
- The Frank aggregation operator is used to define the association between several attributes when Cr-PyFS is present.
- Two instances of developed resilient models are the Cr-PyFFWA and Cr-PyFFWG operators using the CRITIC-WASPAS method. To illustrate the efficacy and legitimacy of the proposed methods, a case study and a few salient characteristics are also discussed.
- Using the data in the context of Cr-PyFFS, an algorithm for the MADM approach is also created. To highlight the efficiency and strength of derived methodologies, we studied an experimental case study to rank different educational institutions under different key criteria and key features. Figure 1 also covers the primary contributions of the manuscript.

### Layout of the manuscript

The remaining parts of the presentation are organized as follows: Section "Methodology" introduces fundamental notions and required concepts for the development of the study, along with recommended methodologies. Section "Circular pythagorean fuzzy frank operations" explores some reliable operations of Frank t-norm and t-conorms under the system of the Cr-PyF context. A list of robust mathematical approaches to Frank t-norm and t-conorm is also developed in Section "Circular pythagorean fuzzy frank aggregation operators". Section "Real-life application for solving MCDM problems" presents a detailed discussion about the CRITIC-WASPAS method to investigate the weight of criteria and ranking of preferences under a system of different key features. A comparative study is also conducted to show the superiority and effectiveness of diaganosed approaches in section "Comparative study". Finally, we illustrate the summary of the article in section "Conclusion".

## Methodology

This part discusses the core concepts of PyFS under the influence of the Frank Aggregation Operator.

### Definition 1

Ref.^50^ Consider a non-empty set $B$. Then, a PyFS $P$ is in the form:

$$P = \left\{\gamma , \left({\mu }_{P}\left(\gamma \right),{\nu }_{P}\left(\gamma \right)\right)|\gamma \in B\right\}$$

where ${\mu }_{P}\left(\gamma \right): B\to \left[\text{0,1}\right]$ and ${\nu }_{P}\left(\gamma \right): B\to \left[\text{0,1}\right]$ denote the membership and non-membership grade of $\gamma$, respectively, provided that $0\le {\mu }_{a}^{2}\left(\gamma \right)+{\nu }_{a}^{2}\left(\gamma \right)\le 1$ and hesitancy degree denoted by ${\pi }_{P}\left(\gamma \right)= \sqrt{1-\left({\mu }_{a}^{2}\left(\gamma \right)+{\nu }_{a}^{2}\left(\gamma \right)\right)}$, ${\pi }_{P}\left(\gamma \right)\in \left[\text{0,1}\right].$

### Definition 2

Ref.^62^ For the universal set $B$, the Cr-PyFS is invented by:

$$A=\left\{ (\gamma , {\mu }_{A}\left(\gamma \right), {v}_{A}\left(\gamma \right), {r}_{A})|\gamma \in B\right\}$$

Note that ${\mu }_{P}\left(\gamma \right): B\to \left[\text{0,1}\right]$ and ${\nu }_{P}\left(\gamma \right): B\to \left[\text{0,1}\right]$ represent the membership and non-membership grade of $\gamma$ in Cr-PyFS. Moreover, the radius $r$ is a point among ${(\mu }_{A}\left(\gamma \right), {v}_{A}\left(\gamma \right))$. The mathematical shape of the Cr-PyFS as $0\le {\mu }_{a}^{2}\left(\gamma \right)+{\nu }_{a}^{2}\left(\gamma \right)\le 1$. Additionally, ${\pi }_{P}\left(\gamma \right)= \sqrt{1-\left({\mu }_{a}^{2}\left(\gamma \right)+{\nu }_{a}^{2}\left(\gamma \right)\right)}$, ${\pi }_{P}\left(\gamma \right)\in \left[\text{0,1}\right]$ indicates the hesitancy value of $\gamma$ in $A$. A circular-pythagorean fuzzy value (Cr-PyFV) is expressed as $\alpha =\left({\mu }_{A}\left(\gamma \right), {v}_{A}\left(\gamma \right):{r}_{A}\right)$.

### Definition 3

Ref.^63^ Let $\alpha$ be a Cr-PyFV and score function ${\mathbb{S}}\left(\alpha \right)$ and accuracy function $A\left(\alpha \right)$ are given by:

1 $${\mathbb{S}}\left(\alpha \right)=\left(\frac{1}{2}\left({\mu }_{ij}^{2}-{v}_{ij}^{2}+\sqrt{2r}\left(\vartheta -1\right)\right)\right), \vartheta \in \left[-\text{1,1}\right]$$

and

2 $$A\left(\alpha \right)=\left({\mu }^{2}\left(x\right)+{v}^{2}\left(x\right), A\left(\alpha \right)\in \left[\text{0,1}\right]\right)$$

### Definition 4

Ref.^63^ Consider any two C-PyFVs, ${\alpha }_{i}=\left({\mu }_{i}, {v}_{i}, {r}_{i}\right) \left(i=1, 2\right)$. Then:

- $${\alpha }_{1}{\oplus }_{t}{\alpha }_{2}=\left({\mu }_{1}^{2}+{\mu }_{2}^{2}-{\mu }_{1}^{2}{\mu }_{2}^{2},{v}_{1}^{2}{v}_{2}^{2}, {r}_{1}+{r}_{2}-{r}_{1}{r}_{2}\right)$$
- $${\alpha }_{1}{\oplus }_{tc}{\alpha }_{2}=\left({\mu }_{1}^{2}+{\mu }_{2}^{2}-{\mu }_{1}^{2}{\mu }_{2}^{2},{v}_{1}^{2}{v}_{2}^{2}, {r}_{1}{r}_{2}\right)$$
- $${\alpha }_{1}{\otimes }_{t}{\alpha }_{2}=\left({\mu }_{1}^{2}{\mu }_{2}^{2}, {v}_{1}^{2}+{v}_{2}^{2}-{v}_{1}^{2}{v}_{2}^{2}, {r}_{1}{r}_{2}\right)$$
- $${\alpha }_{1}{\otimes }_{tc}{\alpha }_{2}=\left({\mu }_{1}^{2}{\mu }_{2}^{2}, {v}_{1}^{2}+{v}_{2}^{2}-{v}_{1}^{2}{v}_{2}^{2},{r}_{1}+{r}_{2}-{r}_{1}{r}_{2}\right)$$
- $$\chi {\alpha }_{{1}_{t}}=(1-(1-{\mu }_{1}^{2}{)}^{\chi }, {\left({v}_{1}^{2}\right)}^{\chi }, 1-\left(1-{r}_{1}{)}^{\chi }\right), \chi >0$$
- $$\chi {\alpha }_{{1}_{tc}}=(1-(1-{\mu }_{1}^{2}{)}^{\chi }, {\left({v}_{1}^{2}\right)}^{\chi }, {r}_{1}^{\chi }), \chi >0$$
- $${\alpha }_{{1}_{t}}^{\chi }={\left({(\mu }_{1}^{2}\right)}^{\chi },1-(1-{v}_{1}^{2}{)}^{\chi }, {r}_{1}^{\chi }), \chi >0$$
- $${\alpha }_{{1}_{tc}}^{\chi }={\left({(\mu }_{1}^{2}\right)}^{\chi }, 1-(1-{v}_{1}^{2}{)}^{\chi },1-\left(1-{r}_{1}{)}^{\chi }\right), \chi >0$$

### Definition 5

Ref.^50^ The theory of Frank t-norm and t-conorm is expressed as follows:

3 $${Fra\left(a,b\right)}_{t}={log}_{\rho }\left(1+\frac{\left({\rho }^{a}-1\right)\left({\rho }^{b}-1\right)}{\rho -1}\right)$$

and

4 $${Fra\left(a,b\right)}_{tc}=1-{log}_{\rho }\left(1+\frac{\left({\rho }^{1-a}-1\right)\left({\rho }^{1-b}-1\right)}{\rho -1}\right)$$

where $$\left(a,b\right)\in \left[\text{0,1}\right]\times [\text{0,1}]$$ and $$\rho \ne 1.$$

## Circular pythagorean fuzzy frank operations

Here, we formulate flexible operations of Frank aggregation operators based on Cr-PyF framework.

### Definition 6

Ref.^64^ Suppose $\alpha =\left({\mu }_{\alpha },{\nu }_{\alpha },{r}_{\alpha }\right), {\alpha }_{1}=\left({{\mu }_{\alpha }}_{1},{{\nu }_{\alpha }}_{1},{{r}_{\alpha }}_{1}\right)$ and ${\alpha }_{2}=\left({{\mu }_{\alpha }}_{2},{{\nu }_{\alpha }}_{2},{{r}_{\alpha }}_{2}\right)$ are the three Cr-PyFVs with real numbers $\rho >1,\chi >0$. Then, we have:

- $${\alpha }_{1}{\oplus }_{min}{\alpha }_{2}=\left(\begin{array}{c}\sqrt{1-{\mathit{log}}_{\rho }\left(1+\frac{\left(\chi {\rho }^{1-{{\mu }_{\alpha }^{2}}_{1}}-1\right)\left({\rho }^{1-{{\mu }_{\alpha }^{2}}_{2}}-1\right)}{\rho -1}\right)}, \\ \sqrt{{\mathit{log}}_{\rho }\left(1+\frac{\left({\rho }^{{{\nu }_{\alpha }^{2}}_{1}}-1\right)\left({\rho }^{{{\nu }_{\alpha }^{2}}_{2}}-1\right)}{\rho -1}\right)},\\ \sqrt{{\mathit{log}}_{k}\left(1+\frac{\left({\rho }^{{{r}_{\alpha }^{2}}_{1}}-1\right)\left({\rho }^{{{r}_{\alpha }^{2}}_{2}}-1\right)}{\rho -1}\right)}\end{array}\right)$$
- $${\alpha }_{1}{\oplus }_{max}{\alpha }_{2}=\left(\begin{array}{c}\sqrt{1-{\mathit{log}}_{\rho }\left(1+\frac{\left({\rho }^{1-{{\mu }_{\alpha }^{2}}_{1}}-1\right)\left({\rho }^{1-{{\mu }_{\alpha }^{2}}_{2}}-1\right)}{\rho -1}\right)}, \\ \sqrt{{\mathit{log}}_{\rho }\left(1+\frac{\left({\rho }^{{{\nu }_{\alpha }^{2}}_{1}}-1\right)\left({\rho }^{{{\nu }_{\alpha }^{2}}_{2}}-1\right)}{\rho -1}\right)},\\ \sqrt{1-{\mathit{log}}_{\rho }\left(1+\frac{\left({\rho }^{1-{{r}_{\alpha }^{2}}_{1}}-1\right)\left({\rho }^{1-{{r}_{\alpha }^{2}}_{2}}-1\right)}{\rho -1}\right)}\end{array}\right)$$
- $${\alpha }_{1}{\otimes }_{min}{\alpha }_{2}=\left(\begin{array}{c}\sqrt{{\mathit{log}}_{\rho }\left(1+\frac{\left({\rho }^{{{\mu }_{\alpha }^{2}}_{1}}-1\right)\left({\rho }^{{{\mu }_{\alpha }^{2}}_{2}}-1\right)}{\rho -1}\right)}, \\ \sqrt{1-{\mathit{log}}_{\rho }\left(1+\frac{\left({\rho }^{1-{{\nu }_{\alpha }^{2}}_{1}}-1\right)\left({\rho }^{1-{{\nu }_{\alpha }^{2}}_{2}}-1\right)}{\rho -1}\right)},\\ \sqrt{1-{\mathit{log}}_{\rho }\left(1+\frac{\left({\rho }^{1-{{r}_{\alpha }^{2}}_{1}}-1\right)\left({\rho }^{1-{{r}_{\alpha }^{2}}_{2}}-1\right)}{\rho -1}\right)}\end{array}\right)$$
- $${\alpha }_{1}{\otimes }_{max}{\alpha }_{2}=\left(\begin{array}{c}\sqrt{{\mathit{log}}_{\rho }\left(1+\frac{\left({\rho }^{{{\mu }_{\alpha }^{2}}_{1}}-1\right)\left({\rho }^{{{\mu }_{\alpha }^{2}}_{2}}-1\right)}{\rho -1}\right)}, \\ \sqrt{1-{\mathit{log}}_{\rho }\left(1+\frac{\left({\rho }^{1-{{\nu }_{\alpha }^{2}}_{1}}-1\right)\left({\rho }^{1-{{\nu }_{\alpha }^{2}}_{2}}-1\right)}{\rho -1}\right)},\\ \sqrt{{\mathit{log}}_{\rho }\left(1+\frac{\left({\rho }^{{{r}_{\alpha }^{2}}_{1}}-1\right)\left({\rho }^{{{r}_{\alpha }^{2}}_{2}}-1\right)}{\rho -1}\right)}\end{array}\right)$$
- $$\chi {\alpha }_{min}=\left(\begin{array}{c}\sqrt{1-{\mathit{log}}_{\rho }\left(1+\frac{{\left({\rho }^{1-{\mu }_{\alpha }^{2}}-1\right)}^{\chi }}{{\left(\rho -1\right)}^{\chi -1}}\right)},\\ \sqrt{{\mathit{log}}_{\rho }\left(1+\frac{{\left({\rho }^{{\nu }_{\alpha }^{2}}-1\right)}^{\chi }}{{\left(\rho -1\right)}^{\chi -1}}\right)},\\ \sqrt{{\mathit{log}}_{k}\left(1+\frac{{\left({\rho }^{{r}_{\alpha }^{2}}-1\right)}^{\chi }}{{\left(\rho -1\right)}^{\chi -1}}\right)}\end{array}\right)$$
- $$\chi {\alpha }_{max}=\left(\begin{array}{c}\sqrt{1-{\mathit{log}}_{\rho }\left(1+\frac{{\left({\rho }^{1-{\mu }_{\alpha }^{2}}-1\right)}^{\chi }}{{\left(\rho -1\right)}^{\chi -1}}\right)},\\ \sqrt{{\mathit{log}}_{\rho }\left(1+\frac{{\left({\rho }^{{\nu }_{\alpha }^{2}}-1\right)}^{\chi }}{{\left(\rho -1\right)}^{\chi -1}}\right)},\\ \sqrt{1-{\mathit{log}}_{\rho }\left(1+\frac{{\left({\rho }^{1-{r}_{\alpha }^{2}}-1\right)}^{\chi }}{{\left(\rho -1\right)}^{\chi -1}}\right)}\end{array}\right)$$
- $${\alpha }_{min}^{\chi }=\left(\begin{array}{c}\sqrt{{\mathit{log}}_{k}\left(1+\frac{{\left({\rho }^{{\mu }_{\alpha }^{2}}-1\right)}^{\chi }}{{\left(\rho -1\right)}^{\chi -1}}\right)},\\ \sqrt{1-{\mathit{log}}_{\rho }\left(1+\frac{{\left({\rho }^{1-{\nu }_{\alpha }^{2}}-1\right)}^{\chi }}{{\left(\rho -1\right)}^{\chi -1}}\right)},\\ \sqrt{1-{\mathit{log}}_{\rho }\left(1+\frac{{\left({\rho }^{1-{r}_{\alpha }^{2}}-1\right)}^{\chi }}{{\left(\rho -1\right)}^{\chi -1}}\right)}\end{array}\right)$$
- $${\alpha }_{max}^{\chi }=\left(\begin{array}{c}\sqrt{{\mathit{log}}_{k}\left(1+\frac{{\left({\rho }^{{\mu }_{\alpha }^{2}}-1\right)}^{\chi }}{{\left(\rho -1\right)}^{\chi -1}}\right)},\\ \sqrt{1-{\mathit{log}}_{\rho }\left(1+\frac{{\left({\rho }^{1-{\nu }_{\alpha }^{2}}-1\right)}^{\chi }}{{\left(\rho -1\right)}^{\chi -1}}\right)},\\ \sqrt{{\mathit{log}}_{k}\left(1+\frac{{\left({\rho }^{{r}_{\alpha }^{2}}-1\right)}^{\chi }}{{\left(\rho -1\right)}^{\chi -1}}\right)}\end{array}\right)$$

### ***Theorem 1***

Consider $\alpha =\left({\mu }_{\alpha },{\nu }_{\alpha },{r}_{\alpha }\right), {\alpha }_{1}=\left({{\mu }_{\alpha }}_{1},{{\nu }_{\alpha }}_{1},{{r}_{\alpha }}_{1}\right)$ and ${\alpha }_{2}=\left({{\mu }_{\alpha }}_{2},{{\nu }_{\alpha }}_{2},{{r}_{\alpha }}_{2}\right)$ are three Cr-PyFVs and $\rho >1, \chi ,{\chi }_{1},{\chi }_{2}>0$ are any real numbers. Then we have:

- $${\alpha }_{1}{\oplus }_{min}{\alpha }_{2}={\alpha }_{2}\oplus {\alpha }_{1}$$
- $${\alpha }_{1}{\otimes }_{min}{\alpha }_{2}={\alpha }_{2}\otimes{\alpha }_{1}$$
- $$\chi \left({\alpha }_{1}{\oplus }_{min}{\alpha }_{2}\right)={\chi \alpha }_{1}\oplus {\chi \alpha }_{2}$$
- $${\chi }_{1}\alpha {\oplus }_{min}{\chi }_{2}\alpha =\left({\chi }_{1}+{\chi }_{2}\right)\alpha$$
- $${\left({\alpha }_{1}{\otimes }_{min}{\alpha }_{2}\right)}^{\chi }={{\alpha }_{1}}^{\chi }\otimes {{\alpha }_{2}}^{\chi }$$
- $${\alpha }^{{\chi }_{1}}{\otimes }_{min}{\alpha }^{{\chi }_{2}}={\alpha }^{{\chi }_{1}+{\chi }_{2}}$$

### Proof

Consider $\alpha =\left({\mu }_{\alpha },{\nu }_{\alpha },{r}_{\alpha }\right), {\alpha }_{1}=\left({{\mu }_{\alpha }}_{1},{{\nu }_{\alpha }}_{1},{{r}_{\alpha }}_{1}\right)$ and ${\alpha }_{2}=\left({{\mu }_{\alpha }}_{2},{{\nu }_{\alpha }}_{2},{{r}_{\alpha }}_{2}\right)$ are three Cr-PyFVs and for any $\chi ,{\chi }_{1},{\chi }_{2}>0$. Then,

$${\alpha }_{1}{\oplus }_{min}{\alpha }_{2}=\left(\begin{array}{c}\sqrt{1-{\mathit{log}}_{\rho }\left(1+\frac{\left({\rho }^{1-{{\mu }_{\alpha }^{2}}_{1}}-1\right)\left({\rho }^{1-{{\mu }_{\alpha }}_{2}}-1\right)}{\rho -1}\right)},\\ \sqrt{{log}_{\rho }\left(1+\frac{\left({\rho }^{{{\nu }_{\alpha }^{2}}_{1}}-1\right)\left({\rho }^{{{\nu }_{\alpha }}_{2}}-1\right)}{\rho -1}\right)},\\ \sqrt{{log}_{\rho }\left(1+\frac{\left({\rho }^{{{r}_{\alpha }^{2}}_{1}}-1\right)\left({\rho }^{{{r}_{\alpha }}_{2}}-1\right)}{\rho -1}\right)}\end{array}\right)$$

$$=\left(\begin{array}{c}\sqrt{1-{\mathit{log}}_{\rho }\left(1+\frac{\left({\rho }^{1-{{\mu }_{\alpha }^{2}}_{2}}-1\right)\left({\rho }^{1-{{\mu }_{\alpha }^{2}}_{1}}-1\right)}{\rho -1}\right)},\\ \sqrt{{log}_{\rho }\left(1+\frac{\left({\rho }^{{{\nu }_{\alpha }^{2}}_{2}}-1\right)\left({\rho }^{{{\nu }_{\alpha }^{2}}_{1}}-1\right)}{\rho -1}\right)},\\ \sqrt{{log}_{\rho }\left(1+\frac{\left({\rho }^{{{r}_{\alpha }^{2}}_{2}}-1\right)\left({\rho }^{{{r}_{\alpha }^{2}}_{1}}-1\right)}{\rho -1}\right)}\end{array}\right)$$

$${=\alpha }_{2}{\oplus }_{min}{\alpha }_{1}$$

$${\alpha }_{1}{\otimes }_{min}{\alpha }_{2}=\left(\begin{array}{c}\sqrt{{log}_{\rho }\left(1+\frac{\left({\rho }^{{{\mu }_{\alpha }^{2}}_{1}}-1\right)\left({\rho }^{{{\mu }_{\alpha }^{2}}_{2}}-1\right)}{\rho -1}\right)},\\ \sqrt{1-{\mathit{log}}_{\rho }\left(1+\frac{\left({\rho }^{1-{{\nu }_{\alpha }^{2}}_{1}}-1\right)\left({\rho }^{1-{{\nu }_{\alpha }^{2}}_{2}}-1\right)}{\rho -1}\right)},\\ \sqrt{1-{\mathit{log}}_{\rho }\left(1+\frac{\left({\rho }^{1-{{r}_{\alpha }^{2}}_{1}}-1\right)\left({\rho }^{1-{{r}_{\alpha }^{2}}_{2}}-1\right)}{\rho -1}\right)}\end{array}\right)$$

$$=\left(\begin{array}{c}\sqrt{{log}_{\rho }\left(1+\frac{\left({\rho }^{{{\mu }_{\alpha }^{2}}_{2}}-1\right)\left({\rho }^{{{\mu }_{\alpha }^{2}}_{1}}-1\right)}{\rho -1}\right)},\\ \sqrt{1-{\mathit{log}}_{\rho }\left(1+\frac{\left({\rho }^{1-{{\nu }_{\alpha }^{2}}_{2}}-1\right)\left({\rho }^{1-{{\nu }_{\alpha }^{2}}_{1}}-1\right)}{\rho -1}\right)},\\ \sqrt{1-{\mathit{log}}_{\rho }\left(1+\frac{\left({\rho }^{1-{{r}_{\alpha }^{2}}_{2}}-1\right)\left({\rho }^{1-{{r}_{\alpha }^{2}}_{1}}-1\right)}{\rho -1}\right)}\end{array}\right)$$

$$={\alpha }_{2}{\otimes }_{min}{\alpha }_{1}$$

$$\chi \left({\alpha }_{1}{\oplus }_{min}{\alpha }_{2}\right)=\chi \left(\begin{array}{c}\sqrt{1-{\mathit{log}}_{\rho }\left(1+\frac{\left({\rho }^{1-{{\mu }_{\alpha }^{2}}_{1}}-1\right)\left({\rho }^{1-{{\mu }_{\alpha }^{2}}_{2}}-1\right)}{\rho -1}\right)},\\ \sqrt{{log}_{\rho }\left(1+\frac{\left({\rho }^{{{\nu }_{\alpha }^{2}}_{1}}-1\right)\left({\rho }^{{{\nu }_{\alpha }^{2}}_{2}}-1\right)}{\rho -1}\right)},\\ \sqrt{{log}_{\rho }\left(1+\frac{\left({\rho }^{{{r}_{\alpha }^{2}}_{1}}-1\right)\left({\rho }^{{{r}_{\alpha }^{2}}_{2}}-1\right)}{\rho -1}\right)}\end{array}\right)$$

$$=\left(\begin{array}{c}\sqrt{1-{\mathit{log}}_{\rho }\left(1+\frac{{\left({\rho }^{1-{{\mu }_{\alpha }^{2}}_{1}}-1\right)}^{\chi }\left({\left({\rho }^{1-{{\mu }_{\alpha }^{2}}_{2}}-1\right)}^{\chi }\right)}{{\left(\rho -1\right)}^{2\chi -1}}\right)},\\ \sqrt{{\mathit{log}}_{\rho }\left(1+\frac{{\left({\rho }^{1-{{\nu }_{\alpha }^{2}}_{1}}-1\right)}^{\chi }\left({\left({\rho }^{1-{{\nu }_{\alpha }^{2}}_{2}}-1\right)}^{\chi }\right)}{{\left(\rho -1\right)}^{2\chi -1}}\right),}\\ \sqrt{{\mathit{log}}_{k}\left(1+\frac{{\left({\rho }^{1-{{r}_{\alpha }^{2}}_{1}}-1\right)}^{\chi }\left({\left({\rho }^{1-{{r}_{\alpha }^{2}}_{2}}-1\right)}^{\chi }\right)}{{\left(\rho -1\right)}^{2\chi -1}}\right)}\end{array}\right)$$

Now

$${\chi \alpha }_{1}{\oplus }_{min}{\chi \alpha }_{2}=\left(\left(\begin{array}{c}\sqrt{{1-\mathit{log}}_{\rho }\left(1+\frac{{\left({\rho }^{1-{{\mu }_{\alpha }^{2}}_{1}}-1\right)}^{\chi }}{{\left(\rho -1\right)}^{\chi }}\right)},\\ \sqrt{{\mathit{log}}_{\rho }\left(1+\frac{{\left({\rho }^{{{\nu }_{\alpha }^{2}}_{1}}-1\right)}^{\chi }}{{\left(\rho -1\right)}^{\chi }}\right),}\\ \sqrt{{\mathit{log}}_{\rho }\left(1+\frac{{\left({\rho }^{{{r}_{\alpha }^{2}}_{1}}-1\right)}^{\chi }}{{\left(\rho -1\right)}^{\chi }}\right)}\end{array}\right) {\oplus }_{min}\left(\begin{array}{c}\sqrt{{1-\mathit{log}}_{\rho }\left(1+\frac{{\left({\rho }^{1-{{\mu }_{\alpha }^{2}}_{2}}-1\right)}^{\chi }}{{\left(\rho -1\right)}^{\chi }}\right)},\\ \sqrt{{\mathit{log}}_{\rho }\left(1+\frac{{\left({\rho }^{{{\nu }_{\alpha }^{2}}_{2}}-1\right)}^{\chi }}{{\left(\rho -1\right)}^{\chi }}\right)},\\ \sqrt{{\mathit{log}}_{\rho }\left(1+\frac{{\left({\rho }^{{{r}_{\alpha }^{2}}_{2}}-1\right)}^{\chi }}{{\left(\rho -1\right)}^{\chi }}\right)}\end{array}\right)\right)$$

$$=\left(\begin{array}{c}\sqrt{1-{\mathit{log}}_{\rho }\left(1+\frac{{\left({\rho }^{1-{{\mu }_{\alpha }^{2}}_{1}}-1\right)}^{\chi }\left({\left({\rho }^{1-{{\mu }_{\alpha }^{2}}_{2}}-1\right)}^{\chi }\right)}{{\left(\rho -1\right)}^{2\chi -1}}\right)},\\ \sqrt{{\mathit{log}}_{\rho }\left(1+\frac{{\left({\rho }^{1-{{\nu }_{\alpha }^{2}}_{1}}-1\right)}^{\chi }\left({\left({\rho }^{1-{{\nu }_{\alpha }^{2}}_{2}}-1\right)}^{\chi }\right)}{{\left(\rho -1\right)}^{2\chi -1}}\right)},\\ \sqrt{{\mathit{log}}_{\rho }\left(1+\frac{{\left({\rho }^{1-{{r}_{\alpha }^{2}}_{1}}-1\right)}^{\chi }\left({\left({\rho }^{1-{{r}_{\alpha }^{2}}_{2}}-1\right)}^{\chi }\right)}{{\left(\rho -1\right)}^{2\chi -1}}\right)}\end{array}\right)$$

$$\chi \left({\alpha }_{1}{\oplus }_{min}{\alpha }_{2}\right)={\chi \alpha }_{1}\oplus {\chi \alpha }_{2}$$

$${\chi }_{1}\alpha {\oplus }_{min}{\chi }_{2}\alpha =\left(\begin{array}{c}\sqrt{1-{\mathit{log}}_{\rho }\left(1+\frac{{\left({\rho }^{1-{\mu }_{\alpha }^{2}}-1\right)}^{{\chi }_{1}}}{{\left(\rho -1\right)}^{{\chi }_{1}}}\right)},\\ \sqrt{{\mathit{log}}_{\rho }\left(1+\frac{{\left({\rho }^{{\nu }_{\alpha }^{2}}-1\right)}^{{\chi }_{1}}}{{\left(\rho -1\right)}^{{\chi }_{1}}}\right), }\\ \sqrt{{\mathit{log}}_{\rho }\left(1+\frac{{\left({\rho }^{{r}_{\alpha }^{2}}-1\right)}^{{\chi }_{1}}}{{\left(\rho -1\right)}^{{\chi }_{1}}}\right)}\end{array}\right)\oplus \left(\begin{array}{c}\sqrt{{1-\mathit{log}}_{\rho }\left(1+\frac{{\left({\rho }^{1-{\mu }_{\alpha }^{2}}-1\right)}^{{\chi }_{2}}}{{\left(\rho -1\right)}^{{\chi }_{2}}}\right), }\\ \sqrt{{\mathit{log}}_{\rho }\left(1+\frac{{\left({\rho }^{{\nu }_{\alpha }^{2}}-1\right)}^{{\chi }_{2}}}{{\left(\rho -1\right)}^{{\chi }_{2}}}\right), }\\ \sqrt{{\mathit{log}}_{\rho }\left(1+\frac{{\left({\rho }^{{r}_{\alpha }^{2}}-1\right)}^{{\chi }_{2}}}{{\left(\rho -1\right)}^{{\chi }_{2}}}\right)}\end{array}\right)$$

$$=\left(\begin{array}{c}\sqrt{{1-\mathit{log}}_{\rho }\left(1+\frac{{\left({\rho }^{1-{\mu }_{\alpha }^{2}}-1\right)}^{{\chi }_{1}+{\chi }_{2}}}{{\left(\rho -1\right)}^{{\chi }_{1+{\chi }_{2}}}}\right)}, \\ \sqrt{{\mathit{log}}_{\rho }\left(1+\frac{{\left({\rho }^{{\nu }_{\alpha }^{2}}-1\right)}^{{\chi }_{1}+{\chi }_{2}}}{{\left(\rho -1\right)}^{{\chi }_{1}+{\chi }_{2}}}\right), }\\ \sqrt{{\mathit{log}}_{\rho }\left(1+\frac{{\left({\rho }^{{r}_{\alpha }^{2}}-1\right)}^{{\chi }_{1}+{\chi }_{2}}}{{\left(\rho -1\right)}^{{\chi }_{1}+{\chi }_{2}}}\right)}\end{array}\right)$$

$$=\left({\chi }_{1}+{\chi }_{2}\right)\alpha$$

$${\left({\alpha }_{1}{\otimes }_{min}{\alpha }_{2}\right)}^{\chi }={\left(\begin{array}{c}\sqrt{{log}_{\rho }\left(1+\frac{\left({\rho }^{{{\mu }_{\alpha }^{2}}_{1}}-1\right)\left({\rho }^{{{\mu }_{\alpha }^{2}}_{2}}-1\right)}{\rho -1}\right)},\\ \sqrt{1-{log}_{\rho }\left(1+\frac{\left({\rho }^{1-{{\nu }_{\alpha }^{2}}_{1}}-1\right)\left({\rho }^{1-{{\nu }_{\alpha }^{2}}_{2}}-1\right)}{\rho -1}\right)},\\ \sqrt{1-{\mathit{log}}_{\rho }\left(1+\frac{\left({\rho }^{1-{{r}_{\alpha }^{2}}_{1}}-1\right)\left({\rho }^{1-{{r}_{\alpha }^{2}}_{2}}-1\right)}{\rho -1}\right)}\end{array}\right)}^{\chi }$$

$$=\left(\begin{array}{c}\sqrt{{log}_{\rho }\left(1+\frac{{\left(\left({\rho }^{{{\mu }_{\alpha }^{2}}_{1}}-1\right)\left({\rho }^{{{\mu }_{\alpha }^{2}}_{2}}-1\right)\right)}^{\chi }}{{\left(\rho -1\right)}^{2\chi -1}}\right)},\\ \sqrt{1-{log}_{\rho }\left(1+\frac{{\left(\left({\rho }^{1-{{\nu }_{\alpha }^{2}}_{1}}-1\right)\left({\rho }^{{1-{\nu }_{\alpha }^{2}}_{2}}-1\right)\right)}^{\chi }}{{\left(\rho -1\right)}^{2\chi -1}}\right)},\\ \sqrt{1-{log}_{\rho }\left(1+\frac{{\left(\left({\rho }^{{1-{r}_{\alpha }^{2}}_{1}}-1\right)\left({\rho }^{{1-{r}_{\alpha }^{2}}_{2}}-1\right)\right)}^{\chi }}{{\left(\rho -1\right)}^{2\chi -1}}\right)}\end{array}\right)$$

$$=\left(\begin{array}{c}\sqrt{{log}_{\rho }\left(1+\frac{{\left(\left({\rho }^{{{\mu }_{\alpha }^{2}}_{1}}-1\right)\right)}^{\chi }}{{\left(\rho -1\right)}^{\chi }}\right)},\\ \sqrt{1-{log}_{\rho }\left(1+\frac{{\left(\left({\rho }^{{1-{\nu }_{\alpha }^{2}}_{1}}-1\right)\right)}^{\chi }}{{\left(\rho -1\right)}^{\chi }}\right)},\\ \sqrt{1-{log}_{\rho }\left(1+\frac{{\left(\left({\rho }^{{1-{r}_{\alpha }^{2}}_{1}}-1\right)\right)}^{\chi }}{{\left(\rho -1\right)}^{\chi }}\right)}\end{array}\right)\otimes \left(\begin{array}{c}\sqrt{{log}_{\rho }\left(1+\frac{{\left(\left({\rho }^{{{\mu }_{\alpha }^{2}}_{2}}-1\right)\right)}^{\chi }}{{\left(\rho -1\right)}^{\chi }}\right)},\\ \sqrt{1-{log}_{\rho }\left(1+\frac{{\left(\left({\rho }^{{1-{\nu }_{\alpha }^{2}}_{2}}-1\right)\right)}^{\chi }}{{\left(\rho -1\right)}^{\chi }}\right)},\\ \sqrt{1-{log}_{\rho }\left(1+\frac{{\left(\left({\rho }^{{1-{r}_{\alpha }^{2}}_{2}}-1\right)\right)}^{\chi }}{{\left(\rho -1\right)}^{\chi }}\right)}\end{array}\right)$$

$$={{\alpha }_{1}}^{\chi }{\otimes }_{min}{{\alpha }_{2}}^{\chi }$$

$${\alpha }^{{\chi }_{1}}{{\otimes }_{min}\alpha }^{{\chi }_{2}}=\left(\begin{array}{c}\sqrt{{\mathit{log}}_{\rho }\left(1+\frac{{\left({\rho }^{{\mu }_{\alpha }^{2}}-1\right)}^{{\chi }_{1}}}{{\left(\rho -1\right)}^{{\chi }_{1}-1}}\right)},\\ \sqrt{1-{log}_{\rho }\left(1+\frac{{\left({\rho }^{1-{\nu }_{\alpha }^{2}}-1\right)}^{{\chi }_{1}}}{{\left(\rho -1\right)}^{{\chi }_{1}-1}}\right)},\\ \sqrt{1-{\mathit{log}}_{\rho }\left(1+\frac{{\left({\rho }^{1-{r}_{\alpha }^{2}}-1\right)}^{{\chi }_{1}}}{{\left(\rho -1\right)}^{{\chi }_{1}-1}}\right)}\end{array}\right)\otimes \left(\begin{array}{c}\sqrt{{\mathit{log}}_{\rho }\left(1+\frac{{\left({\rho }^{{\mu }_{\alpha }^{2}}-1\right)}^{{\chi }_{2}}}{{\left(\rho -1\right)}^{{\chi }_{2}-1}}\right)},\\ \sqrt{1-{log}_{\rho }\left(1+\frac{{\left({\rho }^{1-{\nu }_{\alpha }^{2}}-1\right)}^{{\chi }_{2}}}{{\left(\rho -1\right)}^{{\chi }_{2}-1}}\right)},\\ \sqrt{1-{\mathit{log}}_{\rho }\left(1+\frac{{\left({\rho }^{1-{r}_{\alpha }^{2}}-1\right)}^{{\chi }_{2}}}{{\left(\rho -1\right)}^{{\chi }_{2}-1}}\right)}\end{array}\right)$$

$$=\left(\begin{array}{c}\sqrt{{\mathit{log}}_{\rho }\left(1+\frac{{\left({\rho }^{{\mu }_{\alpha }^{2}}-1\right)}^{{\chi }_{1}+{\chi }_{2}}}{{\left(\rho -1\right)}^{{{\chi }_{1}+\chi }_{2}-1}}\right)},\\ \sqrt{1-{log}_{\rho }\left(1+\frac{{\left({\rho }^{1-{\nu }_{\alpha }^{2}}-1\right)}^{{\chi }_{1}+{\chi }_{2}}}{{\left(\rho -1\right)}^{{{\chi }_{1}+\chi }_{2}-1}}\right)}\\ \sqrt{1-{\mathit{log}}_{\rho }\left(1+\frac{{\left({\rho }^{1-{r}_{\alpha }^{2}}-1\right)}^{{\chi }_{1}+{\chi }_{2}}}{{\left(\rho -1\right)}^{{{\chi }_{1}+\chi }_{2}-1}}\right)}\end{array}\right)$$

$$={\boldsymbol{\Gamma }}^{{{\varvec{\chi}}}_{1}+{{\varvec{\chi}}}_{2}}$$

## Circular pythagorean fuzzy frank aggregation operators

In this section, we desinged robust mathematical approaches of Frank aggregation operators in the light of Cr-PyF circumstances.

### Definition 7

Suppose a set of Cr-PyFVs ${\alpha }_{j}=\left({{\mu }_{\alpha }}_{j},{{\nu }_{\alpha }}_{j},{{r}_{\alpha }}_{j}\right),j=1, 2,\dots , n$. Then, the Cr-PyFFWA operators are expressed as follows:

$$Cr-PyFFW{A}_{min}\left({\alpha }_{1},{\alpha }_{2},\dots ,{\alpha }_{n}\right)=\stackrel{n}{\underset{j=1}{\oplus}}({\varphi }_{j}{\alpha }_{j})$$

5 $$Cr-PyFFW{A}_{max}\left({\alpha }_{1},{\alpha }_{2},\dots ,{\alpha }_{n}\right)=\stackrel{n}{\underset{j=1}{\oplus}}({\varphi }_{j}{\alpha }_{j})$$

### ***Theorem 2***

Suppose a set of Cr-PyFVs ${\alpha }_{j}=\left({{\mu }_{\alpha }}_{j},{{\nu }_{\alpha }}_{j},{{r}_{\alpha }}_{j}\right),j=1, 2,\dots , n$. Then, the integrated value of the Cr-PyFFWA operator is still a PFV, so we have:

6 $$Cr-PyFFW{A}_{min}\left({\alpha }_{1},{\alpha }_{2},\dots ,{\alpha }_{n}\right)=\left(\begin{array}{c}\sqrt{1-{\text{log}}_{\rho }\left(1+\prod_{j=1}^{n}{\left({\rho }^{1-{{\mu }_{\alpha }^{2}}_{j}}-1\right)}^{{\varphi }_{j}}\right)},\\ \sqrt{{\text{log}}_{\rho }\left(1+\prod_{j=1}^{n}{\left({\rho }^{{{\nu }_{\alpha }^{2}}_{j}}-1\right)}^{{\varphi }_{j}}\right)},\\ \sqrt{{\text{log}}_{\rho }\left(1+\prod_{j=1}^{n}{\left({\rho }^{{{r}_{\alpha }^{2}}_{j}}-1\right)}^{{\varphi }_{j}}\right)}\end{array}\right)$$

and

7 $$Cr-PyFFW{A}_{max}\left({\alpha }_{1},{\alpha }_{2},\dots ,{\alpha }_{n}\right)=\left(\begin{array}{c}\sqrt{1-{\text{log}}_{\rho }\left(1+\prod_{j=1}^{n}{\left({\rho }^{1-{{\mu }_{\alpha }^{2}}_{j}}-1\right)}^{{\varphi }_{j}}\right)},\\ \sqrt{{\text{log}}_{\rho }\left(1+\prod_{j=1}^{n}{\left({\rho }^{{{\nu }_{\alpha }^{2}}_{j}}-1\right)}^{{\varphi }_{j}}\right)},\\ \sqrt{1-{\text{log}}_{\rho }\left(1+\prod_{j=1}^{n}{\left({\rho }^{1-{{r}_{\alpha }^{2}}_{j}}-1\right)}^{{\varphi }_{j}}\right)}\end{array}\right)$$

### Proof

Suppose a set of Cr-PyFVs ${\alpha }_{j}=\left({{\mu }_{\alpha }}_{j},{{\nu }_{\alpha }}_{j},{{r}_{\alpha }}_{j}\right),j=1, 2,\dots , n$. We prove the above theorem by using the mathematical induction technique.

**Case 1:** For $$n=2$$

$$Cr-PyFFW{A}_{min}\left({\alpha }_{1}, {\alpha }_{2}\right)=\stackrel{2}{\underset{j=1}{\oplus}}\left({\varphi }_{j}{\alpha }_{j}\right)={\varphi }_{1}{\alpha }_{1}\oplus {\varphi }_{2}{\alpha }_{2}$$

$$=\left(\begin{array}{c}\sqrt{1-{\mathit{log}}_{\rho }\left(1+\frac{{\left({\rho }^{1-{{\mu }_{\alpha }^{2}}_{1}}-1\right)}^{{\varphi }_{1}}}{{\left(\rho -1\right)}^{{\varphi }_{1}-1}}\right),}\\ \sqrt{{\mathit{log}}_{\rho }\left(1+\frac{{\left({\rho }^{{{\nu }_{\alpha }^{2}}_{1}}-1\right)}^{{\varphi }_{1}}}{{\left(\rho -1\right)}^{{\varphi }_{1}-1}}\right),}\\ \sqrt{{\mathit{log}}_{\rho }\left(1+\frac{{\left({\rho }^{{{r}_{\alpha }^{2}}_{1}}-1\right)}^{{\varphi }_{1}}}{{\left(\rho -1\right)}^{{\varphi }_{1}-1}}\right),}\end{array}\right)\oplus$$

$$\left(\begin{array}{c}\sqrt{1-{\mathit{log}}_{\rho }\left(1+\frac{{\left({\rho }^{1-{{\mu }_{\alpha }^{2}}_{2}}-1\right)}^{{\varphi }_{2}}}{{\left(\rho -1\right)}^{{\varphi }_{2}-1}}\right),}\\ \sqrt{{\mathit{log}}_{\rho }\left(1+\frac{{\left({\rho }^{{{\nu }_{\alpha }^{2}}_{2}}-1\right)}^{{\varphi }_{2}}}{{\left(\rho -1\right)}^{{\varphi }_{2}-1}}\right),}\\ \sqrt{{\mathit{log}}_{\rho }\left(1+\frac{{\left({\rho }^{{{r}_{\alpha }^{2}}_{2}}-1\right)}^{{\varphi }_{2}}}{{\left(\rho -1\right)}^{{\varphi }_{2}-1}}\right),}\end{array}\right)$$

$$=\left(\begin{array}{c}\sqrt{1-{\mathit{log}}_{\rho }\left(1+\prod_{j=1}^{2}{\left({\rho }^{1-{{\mu }_{\alpha }^{2}}_{j}}-1\right)}^{{\varphi }_{j}}\right)},\\ \sqrt{{\mathit{log}}_{\rho }\left(1+\prod_{j=1}^{2}{\left({\rho }^{{{\nu }_{\alpha }^{2}}_{j}}-1\right)}^{{\varphi }_{j}}\right)},\\ \sqrt{{\mathit{log}}_{\rho }\left(1+\prod_{j=1}^{2}{\left({\rho }^{{{r}_{\alpha }^{2}}_{j}}-1\right)}^{{\varphi }_{j}}\right)}\end{array}\right)$$

Since the result is proved for $n=2$.

Now, we have to prove the given result holds for $n=t$.

$$Cr-PyFFW{A}_{min} \left({\alpha }_{1}, {\alpha }_{2},\dots ,{\alpha }_{n}\right)=\stackrel{t}{\underset{j=1}{\oplus}}\left({\varphi }_{j}{\alpha }_{j}\right)=\left(\begin{array}{c}\sqrt{1-{\mathit{log}}_{\rho }\left(1+\prod_{j=1}^{t}{\left({\rho }^{1-{{\mu }_{\alpha }^{2}}_{j}}-1\right)}^{{\varphi }_{j}}\right)},\\ \sqrt{{\mathit{log}}_{\rho }\left(1+\prod_{j=1}^{t}{\left({\rho }^{{{\nu }_{\alpha }^{2}}_{j}}-1\right)}^{{\varphi }_{j}}\right)},\\ \sqrt{{\mathit{log}}_{\rho }\left(1+\prod_{j=1}^{t}{\left({\rho }^{{{r}_{\alpha }^{2}}_{j}}-1\right)}^{{\varphi }_{j}}\right)}\end{array}\right)$$

Now, we have to prove Eq. 4 is true for $n=t+1$.

$$Cr-PyFFW{A}_{min}\left({\alpha }_{1}, {\alpha }_{2},\dots ,{\alpha }_{n}\right)=\stackrel{t+1}{\underset{j=1}{\oplus}}\left({\varphi }_{j}{\alpha }_{j}\right)=\stackrel{t}{\underset{j=1}{\oplus}}\left({\varphi }_{j}{\alpha }_{j}\right)\oplus {\varphi }_{t+1}{\alpha }_{t+1}$$

$$=\left(\begin{array}{c}\sqrt{1-{\mathit{log}}_{\rho }\left(1+\frac{\prod_{j=1}^{t}{\left({\rho }^{1-{{\mu }_{\alpha }^{2}}_{j}}-1\right)}^{{\varphi }_{j}}}{{\left(\rho -1\right)}^{\sum_{j=1}^{t}{\varphi }_{j}-1}}\right),}\\ \sqrt{{\mathit{log}}_{\rho }\left(1+\frac{\prod_{j=1}^{t}{\left({\rho }^{{{\nu }_{\alpha }^{2}}_{j}}-1\right)}^{{\varphi }_{j}}}{{\left(\rho -1\right)}^{\sum_{j=1}^{t}{\varphi }_{j}-1}}\right),}\\ \sqrt{{\mathit{log}}_{\rho }\left(1+\frac{\prod_{j=1}^{t}{\left({\rho }^{{{r}_{\alpha }^{2}}_{j}}-1\right)}^{{\varphi }_{j}}}{{\left(\rho -1\right)}^{\sum_{j=1}^{t}{\varphi }_{j}-1}}\right),}\end{array}\right)\oplus \left(\begin{array}{c}\sqrt{1-{\mathit{log}}_{\rho }\left(1+\frac{{\left({\rho }^{1-{{\mu }_{\alpha }^{2}}_{t+1}}-1\right)}^{{\varphi }_{t+1}}}{{\left(\rho -1\right)}^{{\varphi }_{t+1}-1}}\right),}\\ \sqrt{{\mathit{log}}_{\rho }\left(1+\frac{{\left({\rho }^{{{\nu }_{\alpha }^{2}}_{t+1}}-1\right)}^{{\varphi }_{t+1}}}{{\left(\rho -1\right)}^{{\varphi }_{t+1}-1}}\right),}\\ \sqrt{{\mathit{log}}_{\rho }\left(1+\frac{{\left({\rho }^{{{r}_{\alpha }^{2}}_{t+1}}-1\right)}^{{\varphi }_{t+1}}}{{\left(\rho -1\right)}^{{\varphi }_{t+1}-1}}\right),}\end{array}\right)$$

$$=\left(\begin{array}{c}\sqrt{1-{\mathit{lo}g}_{\rho }\left(1+\prod_{j=1}^{t+1}{\left({\rho }^{1-{{\mu }_{\alpha }^{2}}_{j}}-1\right)}^{{\varphi }_{j}}\right)},\\ \sqrt{{\mathit{log}}_{\rho }\left(1+\prod_{j=1}^{t+1}{\left({\rho }^{{{\nu }_{\alpha }^{2}}_{j}}-1\right)}^{{\varphi }_{j}}\right)},\\ \sqrt{{\mathit{log}}_{\rho }\left(1+\prod_{j=1}^{t+1}{\left({\rho }^{{{r}_{\alpha }^{2}}_{j}}-1\right)}^{{\varphi }_{j}}\right)}\end{array}\right)$$

Hence, the above result is true for $n=t+1.$

### ***Theorem 3***

Suppose a set of Cr-PyFVs ${\alpha }_{j}=\left({{\mu }_{\alpha }}_{j},{{\nu }_{\alpha }}_{j},{{r}_{\alpha }}_{j}\right),j=1, 2,\dots , n$ such that ${\alpha }_{j}=\alpha$. Then we have:

$$Cr-PyFFW{A}_{min}\left({\alpha }_{1}, {\alpha }_{2},\dots ,{\alpha }_{n}\right)=\alpha$$

### Proof

Suppose a set of Cr-PyFVs ${\alpha }_{j}=\left({{\mu }_{\alpha }}_{j},{{\nu }_{\alpha }}_{j},{{r}_{\alpha }}_{j}\right),j=1, 2,\dots , n$, so we have:

$$Cr-PyFFW{A}_{min}\left({\alpha }_{1}, {\alpha }_{2},\dots ,{\alpha }_{n}\right)=\left(\begin{array}{c}\sqrt{1-{\mathit{log}}_{\rho }\left(1+\prod_{j=1}^{n}{\left({\rho }^{1-{{\mu }_{\alpha }^{2}}_{j}}-1\right)}^{{\varphi }_{j}}\right)},\\ \sqrt{{\mathit{log}}_{\rho }\left(1+\prod_{j=1}^{n}{\left({\rho }^{{{\nu }_{\alpha }^{2}}_{j}}-1\right)}^{{\varphi }_{j}}\right)},\\ \sqrt{{\mathit{log}}_{\rho }\left(1+\prod_{j=1}^{n}{\left({\rho }^{{{r}_{\alpha }^{2}}_{j}}-1\right)}^{{\varphi }_{j}}\right)}\end{array}\right)$$

$$=\left(\begin{array}{c}\sqrt{1-{\mathit{log}}_{\rho }\left(1+\prod_{j=1}^{n}{\left({\rho }^{1-{\mu }_{\alpha }^{2}}-1\right)}^{{\varphi }_{j}}\right)},\\ \sqrt{{\mathit{log}}_{\rho }\left(1+\prod_{j=1}^{n}{\left({\rho }^{{\nu }_{\alpha }^{2}}-1\right)}^{{\varphi }_{j}}\right)},\\ \sqrt{{\mathit{log}}_{\rho }\left(1+\prod_{j=1}^{n}{\left({\rho }^{{r}_{\alpha }^{2}}-1\right)}^{{\varphi }_{j}}\right)}\end{array}\right)$$

$$=\left(\begin{array}{c}\sqrt{1-{log}_{\rho }\left(1+{\left({\rho }^{1-{\mu }_{\alpha }^{2}}-1\right)}^{\sum_{j=1}^{n}{\varphi }_{j}}\right)},\\ \sqrt{{log}_{\rho }\left(1+{\left({\rho }^{{\nu }_{\alpha }^{2}}-1\right)}^{\sum_{j=1}^{n}{\varphi }_{j}}\right)},\\ \sqrt{{log}_{\rho }\left(1+{\left({\rho }^{{r}_{\alpha }^{2}}-1\right)}^{\sum_{j=1}^{n}{\varphi }_{j}}\right)}\end{array}\right)$$

$$=\left(\begin{array}{c}\sqrt{1-{\mathit{log}}_{\rho }\left(1+\left({\rho }^{1-{\mu }_{\alpha }^{2}}-1\right)\right)},\\ \sqrt{{\mathit{log}}_{\rho }\left(1+\left({\rho }^{{\nu }_{\alpha }^{2}}-1\right)\right)},\\ \sqrt{{\mathit{log}}_{\rho }\left(1+\left({\rho }^{{r}_{\alpha }^{2}}-1\right)\right)}\end{array}\right)$$

$$=\left({\mu }_{\alpha },{\nu }_{\alpha },{r}_{\alpha }\right)=\alpha$$

### ***Theorem 4***

Consider ${\alpha }_{j}=\left({\mu }_{{\alpha }_{j}},{\nu }_{{\alpha }_{j}},{r}_{{\alpha }_{j}}\right)$,$j=1, 2, 3,\dots ,n$ be the assemblage of Cr-PyFVs. Let ${\alpha }^{-}=min\left\{{\alpha }_{1},{\alpha }_{2},\dots ,{\alpha }_{n}\right\}=\left(\mathit{min}\left\{{\mu }_{{\alpha }_{j}}\right\},\mathit{max}\left\{{\nu }_{{\alpha }_{j}}\right\},\mathit{max}\left\{{{r}_{\alpha }}_{j}\right\}\right)$ and ${\alpha }^{+}=max\left\{{\alpha }_{1},{\alpha }_{2},\dots ,{\alpha }_{n}\right\}=\left(\mathit{max}\left\{{\mu }_{{\alpha }_{j}}\right\},\mathit{min}\left\{{\nu }_{{\alpha }_{j}}\right\},\mathit{min}\left\{{{r}_{\alpha }}_{j}\right\}\right)$. Then we have:

$${\alpha }^{-}\le Cr-PyFFW{A}_{min}\left({\alpha }_{1},{\alpha }_{2},\dots ,{\alpha }_{n}\right)\le {\alpha }^{+}$$

### Proof

Consider ${\alpha }_{j}=\left({\mu }_{{\alpha }_{j}},{\nu }_{{\alpha }_{j}},{r}_{{\alpha }_{j}}\right), j=1, 2, 3,\dots ,n$ be the assemblage of Cr-PyFVs. Let ${\alpha }^{-}=min\left\{{\alpha }_{1},{\alpha }_{2},\dots ,{\alpha }_{n}\right\}=\left(\mathit{min}\left\{{\mu }_{{\alpha }_{j}}\right\},\mathit{max}\left\{{\nu }_{{\alpha }_{j}}\right\},\mathit{max}\left\{{{r}_{\alpha }}_{j}\right\}\right)and {\alpha }^{+}=max\left\{{\alpha }_{1},{\alpha }_{2},\dots ,{\alpha }_{n}\right\}=\left(\mathit{max}\left\{{\mu }_{{\alpha }_{j}}\right\},\mathit{min}\left\{{\nu }_{{\alpha }_{j}}\right\},\mathit{min}\left\{{{r}_{\alpha }}_{j}\right\}\right)$. Then we have:

$${\mu }^{-}=\mathit{mi}{n}_{j}\left\{{{\mu }_{\alpha }}_{j}\right\},{\nu }^{-}=\mathit{ma}{x}_{j}\left\{{{\nu }_{\alpha }}_{j}\right\},{r}^{-}=\mathit{ma}{x}_{j}\left\{{{r}_{\alpha }}_{j}\right\}$$

And

$${\mu }^{+}=\mathit{ma}{x}_{j}\left\{{{\mu }_{\alpha }}_{j}\right\},{\nu }^{+}=\mathit{mi}{n}_{j}\left\{{{\nu }_{\alpha }}_{j}\right\}$$

and

$${r}^{+}=\mathit{mi}{n}_{j}\left\{{{r}_{\alpha }}_{j}\right\}.$$

Now,

$$\sqrt{1-{\mathit{log}}_{\rho }\left(1+\prod_{j=1}^{n}{\left({\rho }^{1-{{\mu }^{2}}^{-}}-1\right)}^{{\varphi }_{j}}\right)}\le \sqrt{1-{\mathit{log}}_{\rho }\left(1+\prod_{j=1}^{n}{\left({\rho }^{1-{{\mu }_{\alpha }^{2}}_{j}}-1\right)}^{{\varphi }_{j}}\right)}\le \sqrt{1-{\mathit{log}}_{\rho }\left(1+\prod_{j=1}^{n}{\left({\rho }^{1-{{\mu }^{2}}^{+}}-1\right)}^{{\varphi }_{j}}\right),}$$

$$\sqrt{{\mathit{log}}_{\rho }\left(1+\prod_{j=1}^{n}{\left({\rho }^{{{\nu }^{2}}^{+}}-1\right)}^{{\varphi }_{j}}\right)}\le \sqrt{{\mathit{log}}_{\rho }\left(1+\prod_{j=1}^{n}{\left({\rho }^{{{\nu }_{\alpha }^{2}}_{j}}-1\right)}^{{\varphi }_{j}}\right)}\le \sqrt{{\mathit{log}}_{\rho }\left(1+\prod_{j=1}^{n}{\left({\rho }^{{{\nu }^{2}}^{-}}-1\right)}^{{\varphi }_{j}}\right),}$$

$$\sqrt{{\mathit{log}}_{\rho }\left(1+\prod_{j=1}^{n}{\left({\rho }^{{{r}^{2}}^{+}}-1\right)}^{{\varphi }_{j}}\right)}\le \sqrt{{\mathit{log}}_{\rho }\left(1+\prod_{j=1}^{n}{\left({\rho }^{{{r}_{\alpha }^{2}}_{j}}-1\right)}^{{\varphi }_{j}}\right)}\le \sqrt{{\mathit{log}}_{\rho }\left(1+\prod_{j=1}^{n}{\left({\rho }^{{{r}^{2}}^{-}}-1\right)}^{{\varphi }_{j}}\right),}$$

From this, we concluded:

$${\alpha }^{-}\le Cr-PyFFW{A}_{min}\left({\alpha }_{1},{\alpha }_{2},\dots ,{\alpha }_{n}\right)\le {\alpha }^{+}$$

### ***Theorem 5***

Consider ${\alpha }_{j}=\left({\mu }_{{\alpha }_{j}},{\nu }_{{\alpha }_{j}},{r}_{{\alpha }_{j}}\right)$ and ${\alpha }_{j}{\prime}=\left({\mu }_{{\alpha }_{j}}{\prime},{\nu }_{{\alpha }_{j}}{\prime},{r}_{{\alpha }_{j}}{\prime}\right),j,j=\text{1,2},\dots , n$ be any two sets of Cr-PyFVs. If ${\alpha }_{j}\le {\alpha }_{j}{\prime}$ such that ${\mu }_{{\alpha }_{j}}\le {\mu }_{{\alpha }_{j}}{\prime}, {\nu }_{{\alpha }_{j}}\ge {\nu }_{{\alpha }_{j}}{\prime}$ and ${r}_{{\alpha }_{j}}\ge {r}_{{\alpha }_{j}}{\prime}$. Then we have:

$$Cr-PyFFW{A}_{min}\left({\alpha }_{1},{\alpha }_{2},\dots ,{\alpha }_{n}\right)\le Cr-PyFFW{A}_{min}\left({\alpha }_{1}{\prime},{\alpha }_{2}{\prime},\dots ,{\alpha }_{n}{\prime}\right)$$

### Proof

Since ${\alpha }_{j}=\left({\mu }_{{\alpha }_{j}},{\nu }_{{\alpha }_{j}},{r}_{{\alpha }_{j}}\right)$ and ${\alpha }_{j}{\prime}=\left({\mu }_{{\alpha }_{j}}{\prime},{\nu }_{{\alpha }_{j}}{\prime},{r}_{{\alpha }_{j}}{\prime}\right),j,j=\text{1,2},\dots , n$ be any two sets of Cr-PyFVs. If ${\alpha }_{j}\le {\alpha }_{j}{\prime}$ such that ${\mu }_{{\alpha }_{j}}\le {\mu }_{{\alpha }_{j}}{\prime}, {\nu }_{{\alpha }_{j}}\ge {\nu }_{{\alpha }_{j}}{\prime}$ and ${r}_{{\alpha }_{j}}\ge {r}_{{\alpha }_{j}}{\prime}$. Then we have:

$${\left({\rho }^{1-{\mu }_{{\alpha }_{j}}}-1\right)}^{{\varphi }_{j}}\ge {\left({\rho }^{1-{\mu }_{{\alpha }_{j}}{\prime}}-1\right)}^{{\varphi }_{j}}$$

$$=>{\mathit{log}}_{\rho }\left(1+\prod_{j=1}^{n}{\left({\rho }^{1-{{\mu }_{\alpha }}_{j}}-1\right)}^{{\varphi }_{j}}\right)\ge {\mathit{log}}_{\rho }\left(1+\prod_{j=1}^{n}{\left({\rho }^{1-{{\mu }_{\alpha }{\prime}}_{j}}-1\right)}^{{\varphi }_{j}}\right)$$

$$=>1-{\mathit{log}}_{\rho }\left(1+\prod_{j=1}^{n}{\left({\rho }^{1-{{\mu }_{\alpha }}_{j}}-1\right)}^{{\varphi }_{j}}\right)\le 1-{\mathit{log}}_{\rho }\left(1+\prod_{j=1}^{n}{\left({\rho }^{1-{{\mu }_{\alpha }{\prime}}_{j}}-1\right)}^{{\varphi }_{j}}\right)$$

$$\sqrt{1-{\mathit{log}}_{\rho }\left(1+\prod_{j=1}^{n}{\left({\rho }^{1-{{\mu }_{\alpha }^{2}}_{j}}-1\right)}^{{\varphi }_{j}}\right)}\le \sqrt{1-{\mathit{log}}_{\rho }\left(1+\prod_{j=1}^{n}{\left({\rho }^{1-{{{\mu }^{2}}_{\alpha }{\prime}}_{j}}-1\right)}^{{\varphi }_{j}}\right)}$$

In the same way, we can prove that:

$$\sqrt{{\mathit{log}}_{\rho }\left(1+\prod_{j=1}^{n}{\left({\rho }^{{{\nu }_{\alpha }^{2}}_{j}}-1\right)}^{{\varphi }_{j}}\right)}\ge \sqrt{{\mathit{log}}_{\rho }\left(1+\prod_{j=1}^{n}{\left({\rho }^{1-{{{\nu }^{2}}_{\alpha }{\prime}}_{j}}-1\right)}^{{\varphi }_{j}}\right)}$$

and,

$$\sqrt{{\mathit{log}}_{\rho }\left(1+\prod_{j=1}^{n}{\left({\rho }^{{{r}_{\alpha }^{2}}_{j}}-1\right)}^{{\varphi }_{j}}\right)}\ge \sqrt{{\mathit{log}}_{\rho }\left(1+\prod_{j=1}^{n}{\left({\rho }^{1-{{{r}^{2}}_{\alpha }{\prime}}_{j}}-1\right)}^{{\varphi }_{j}}\right)}$$

Hence,

$$Cr-PyFFW{A}_{min}\left({\alpha }_{1},{\alpha }_{2},\dots ,{\alpha }_{n}\right)\le Cr-PyFFW{A}_{min}\left({\alpha }_{1}{\prime},{\alpha }_{2}{\prime},\dots ,{\alpha }_{n}{\prime}\right)$$

### Definition 8

Suppose a set of Cr-PyFVs ${\alpha }_{j}=\left({{\mu }_{\alpha }}_{j},{{\nu }_{\alpha }}_{j},{{r}_{\alpha }}_{j}\right),j=1, 2,\dots , n$. Then, the Cr-PyFFWG operators are given below:

$$Cr-PyFFW{G}_{min}\left({\alpha }_{1},{\alpha }_{2},\dots ,{\alpha }_{n}\right)=\stackrel{n}{\underset{j=1}{\oplus}}({\varphi }_{j}{\alpha }_{j})$$

8 $$Cr-PyFFW{G}_{max}\left({\alpha }_{1},{\alpha }_{2},\dots ,{\alpha }_{n}\right)=\stackrel{n}{\underset{j=1}{\oplus}}({\varphi }_{j}{\alpha }_{j})$$

### ***Theorem 6***

Suppose a set of Cr-PyFVs ${\alpha }_{j}=\left({{\mu }_{\alpha }}_{j},{{\nu }_{\alpha }}_{j},{{r}_{\alpha }}_{j}\right),j=1, 2,\dots , n$. Then, the integrated value of the Cr-PyFFWG operators is still a Cr-PyFV, so we have:

9 $$Cr-PyFFW{G}_{min}\left({\alpha }_{1},{\alpha }_{2},\dots ,{\alpha }_{n}\right)=\left(\begin{array}{c}\sqrt{{\text{log}}_{\rho }\left(1+\prod_{j=1}^{n}{\left({\rho }^{{{\mu }_{\alpha }^{2}}_{j}}-1\right)}^{{\varphi }_{j}}\right)},\\ \sqrt{1-{\text{log}}_{\rho }\left(1+\prod_{j=1}^{n}{\left({\rho }^{1-{{\nu }_{\alpha }^{2}}_{j}}-1\right)}^{{\varphi }_{j}}\right)},\\ \sqrt{{\text{log}}_{\rho }\left(1+\prod_{j=1}^{n}{\left({\rho }^{{{r}_{\alpha }^{2}}_{j}}-1\right)}^{{\varphi }_{j}}\right)}\end{array}\right)$$

and

10 $$Cr-PyFFW{G}_{max}\left({\alpha }_{1},{\alpha }_{2},\dots ,{\alpha }_{n}\right)=\left(\begin{array}{c}\sqrt{{\text{log}}_{\rho }\left(1+\prod_{j=1}^{n}{\left({\rho }^{{{\mu }_{\alpha }^{2}}_{j}}-1\right)}^{{\varphi }_{j}}\right)},\\ \sqrt{1-{\text{log}}_{\rho }\left(1+\prod_{j=1}^{n}{\left({\rho }^{1-{{\nu }_{\alpha }^{2}}_{j}}-1\right)}^{{\varphi }_{j}}\right)},\\ \sqrt{1-{\text{log}}_{\rho }\left(1+\prod_{j=1}^{n}{\left({\rho }^{1-{{r}_{\alpha }^{2}}_{j}}-1\right)}^{{\varphi }_{j}}\right)}\end{array}\right)$$

## Real-life application for solving MCDM problems

A small number of independent and interdependent attributes are evaluated either qualitatively or statistically before possibilities are ranked using the multi-attribute group decision-making (MAGDM) approach. The goal of this strategy is to solve issues in challenging and uncertain situations.

The CRITIC-WASPAS method plays a significant role in multi-criteria decision-making (MCDM) by effectively integrating objective weighting and robust evaluation of alternatives. The CRITIC (CRiteria Importance Through Intercriteria Correlation) method determines the objective weights of criteria based on the contrast intensity and the conflict (or correlation) among criteria. This ensures that the importance of each criterion is derived from the data itself, reducing subjective bias. Once the criteria weights are obtained, the WASPAS (Weighted Aggregated Sum Product Assessment) method is employed to rank the alternatives. WASPAS combines the strengths of both the Weighted Sum Model (WSM) and the Weighted Product Model (WPM), balancing additive and multiplicative approaches to enhance accuracy and reliability in ranking. Together, CRITIC-WASPAS offers a comprehensive and systematic framework that supports decision-makers in evaluating complex problems with multiple conflicting criteria by providing an objective and well-justified ranking of options.

**Fig. 2:**
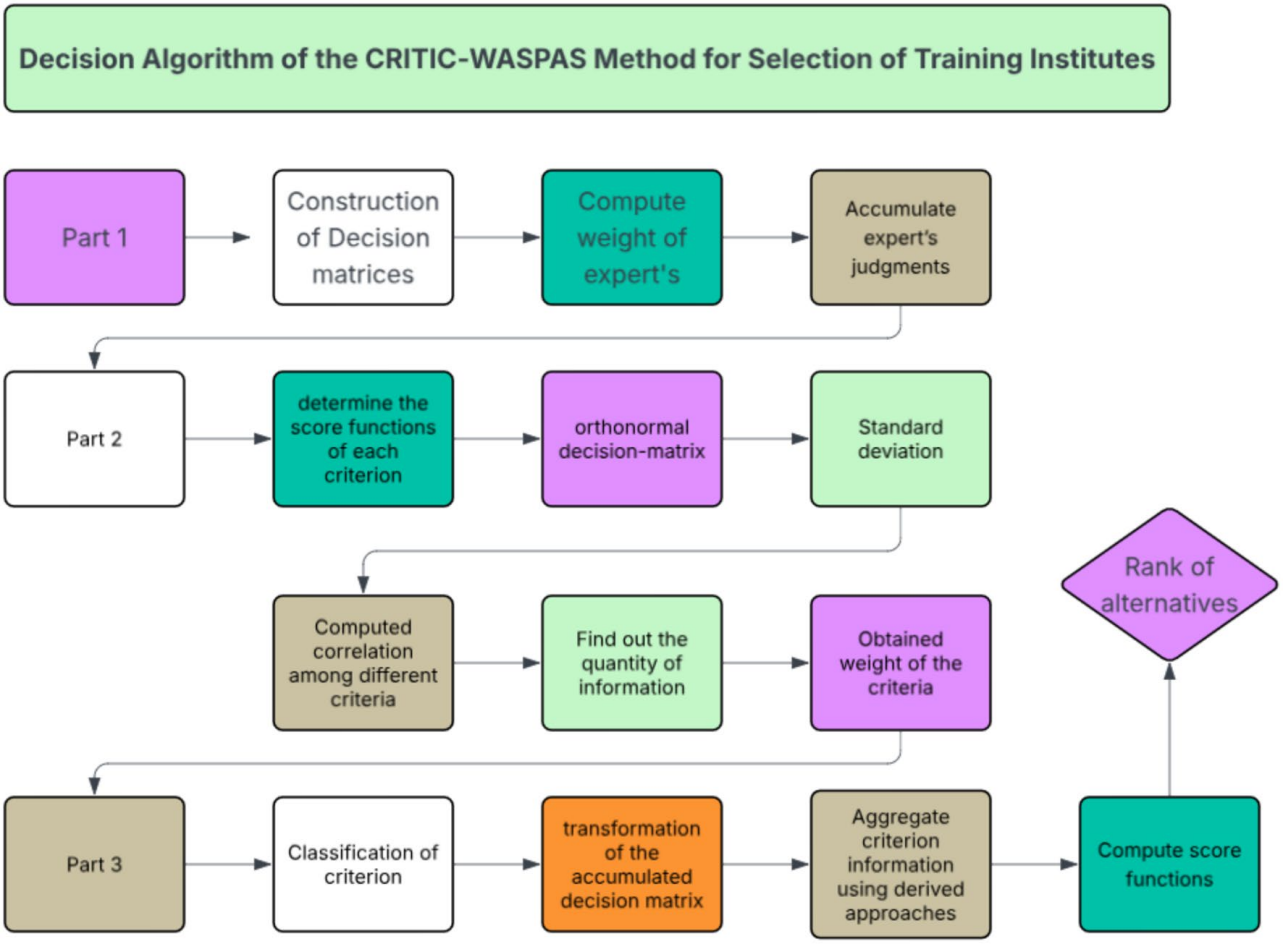
The Diagram explores the stepwise decision algorithm of the CRITIC-WASPAS method.

The MAGDM technique procedure, while accounting for data, is described in this section. Along with the small number of options $G=\left\{{G}_{1},{G}_{2}, {G}_{3},\dots , {G}_{n}\right\}$, the set of attributes $H=\left\{{H}_{1},{H}_{2}, {H}_{3},\dots , {H}_{n}\right\}$ is taken into account. The information on each attribute for each alternative is provided in the form of Cr-PyF ${\chi }_{ij}=\left({\mu }_{ij}, {v}_{ij},{r}_{ij}\right), i=\text{1,2},...,n \& j=\text{1,2},\dots ,m$ where ${\mu }_{ij},{v}_{ij}\in [\text{0,1}]$. We will use the PyF Frank operator to analyze the optimal option based on the MADM problem technique. The Diagram of Fig. 2 also explore the stepwise decision algorithm of the proposed decision-making model. A list of the basic algorithmic steps is as follows.

### Algorithm

To use specific criteria or attributes in order to analyze a prevailing ideal alternative using the MADM problem algorithm.

#### Part 1

**Step 1:** Decision-makers or experts organize their opinions in the form of Cr-PyFVs. This information extensively expresses data about various attributes corresponding to each alternative.

**Step 2:** Determine the weight of the expert using linguistic scales of Cr-PyFVs depicted in Table 1 and the following expression.

**Table 1:** Linguistic scales for weights of experts.

Details	Cr-PyFVs		
Very very important (VVI)	$0.91$	$0.07$	$0.41$
very important (VI)	$0.78$	$0.18$	$0.60$
Important (I)	$0.6$	$0.25$	$0.76$
Not Important (NI)	$0.35$	$0.73$	$0.59$
Very not Important (VNI)	$0.15$	$0.85$	$0.50$

11 $${\text{O}}_{i}=\frac{\left({\mu }_{i}+{\pi }_{i}\left(\frac{{\mu }_{i}}{{\mu }_{i}+{v}_{i}}\right)\right)}{\sum_{i=1}^{s}\left({\mu }_{i}+{\pi }_{i}\left(\frac{{\mu }_{i}}{{\mu }_{i}+{v}_{i}}\right)\right)}$$

**Step 3:** Integrate the expert’s judgment employing the obtained weights of experts and judgments in the form of the Cr-PyF framework.

#### Part 2

There are many methods or techniques used to demonstrate the weights of the criteria. However, we adopted a novel theory of the CRITIC method to investigate the weights of criteria under the following steps.

**Step (a):** First of all, determine the score functions of each criterion associated with alternatives by applying the following formula:

12 $$\delta ={\mathbb{S}}=\left(\frac{1}{2}\left({\mu }_{ij}^{2}-{v}_{ij}^{2}+\sqrt{2r}\left(\vartheta -1\right)\right)\right), \vartheta \in \left[\text{0,1}\right]$$

**Step (b):** Investigate Cr-PyF orthonormal decision-matrix based on score functions as follows:

13 $${K}{\prime}=\left[{\delta }_{ij}{\prime}\right]=\left\{\begin{array}{cc}\frac{{\delta }_{ij}-{\delta }_{j}^{-}}{{\delta }_{j}^{+}-{\delta }_{j}^{-}}& j\in B \left(beneficial type\right)\\ \frac{{\delta }_{j}^{+}-{\delta }_{ij}}{{\delta }_{j}^{+}-{\delta }_{j}^{-}}& j\in C\left(Cost type\right)\end{array}\right.$$

Note that ${\delta }_{j}^{+}=\text{max}\left\{{\delta }_{ij}\right\}$ and ${\delta }_{j}^{-}=\text{min}\left\{{\delta }_{ij}\right\}$.

**Step (c):** Standard deviation obtained based on the following expression:

14 $${\sigma }_{j}=\sqrt{\frac{\sum_{i=1}^{n}{\left({\delta }_{ij}{\prime}-\overline{{\delta }_{i}}\right)}^{2}}{n}}$$

As $$\overline{{\delta }_{j}}=\frac{\sum_{i=1}^{n}{\delta }_{ij}{\prime}}{n}$$.

**Step (d):** Computed correlation among different pairs of criteria employing the following formula:

15 $${\theta }_{jk}=\frac{\sum_{i=1}^{n}\left({\delta }_{ij}{\prime}-\overline{{\delta }_{j}}\right)\left({\delta }_{ij}{\prime}-\overline{{\delta }_{k}}\right)}{\sqrt{\sum_{i=1}^{n}{\left({\delta }_{ij}{\prime}-\overline{{\delta }_{j}}\right)}^{2}\sum_{i=1}^{n}{\left({\delta }_{ij}{\prime}-\overline{{\delta }_{k}}\right)}^{2}}}$$

**Step (e):** Find out the quantity of information associated with the criteria as follows:

16 $${C}_{j}={\sigma }_{j}\sum_{k=1}^{n}\left(1-{\theta }_{jk}\right)$$

**Step (f):** Finally, obtain the weight of the criteria using the following formula:

17 $${\delta }_{j}=\frac{{C}_{j}}{\sum_{j=1}^{n}{C}_{j}}$$

#### Part 3

Based on the robustness and efficiency of the WASPAS method, different research scholars apply to investigate the ranking of preferences, taking into account different fuzzy environments and key criteria. Keeping in the significance of the WASPAS method, we established an intelligent decision algorithm for the WASPAS method under the system of Cr-PyF circumstances.

**Step (a):** Classification of the criterion using the following expressions:

18 $${\alpha }_{ij}=\left(max{\mu }_{ij},min{v}_{ij}, min{r}_{ij}\right), i,j=\text{1,2},\dots , n,m$$

and

19 $${\alpha }_{ij}=\left(min{\mu }_{ij},max{v}_{ij},max{r}_{ij}\right), i,j=\text{1,2},\dots , n,m$$

where $\left(max{\mu }_{ij},min{v}_{ij}, min{r}_{ij}\right)$ and $\left(min{\mu }_{ij},max{v}_{ij},max{r}_{ij}\right)$ expressions used for beneficial and non-beneficial attributes.

The transformation of the accumulated decision matrix is obtained under the following expressions:

20 $${\alpha {\prime}}_{ij}=\left({\mu }_{ij}, {v}_{ij}\right)= \left\{\begin{array}{c}0 otherwise\\ \frac{{\mu }_{ij}}{1+{\mu }_{ij}} of {\mu }_{ij}\le {\mu }_{ij} \\ \frac{{v}_{ij}}{1+{v}_{ij}} for non-membership\end{array}\right.$$

**Step (c):** Aggregate criterion information corresponding to each alternative by employing the proposed methodologies of Cr-PyFFWA and Cr-PyFFWG operators as follows:

21 $${\beta }_{i}^{WSA}=Cr-PyFFW{A}_{min}\left({\alpha }_{1},{\alpha }_{2},\dots ,{\alpha }_{n}\right)=\left(\begin{array}{c}\sqrt{1-{\text{log}}_{\rho }\left(1+\prod_{j=1}^{n}{\left({\rho }^{1-{{\mu }_{\alpha }^{2}}_{ij}}-1\right)}^{{\varphi }_{j}}\right)},\\ \sqrt{{\text{log}}_{\rho }\left(1+\prod_{j=1}^{n}{\left({\rho }^{{{\nu }_{\alpha }^{2}}_{ij}}-1\right)}^{{\varphi }_{j}}\right)},\\ \sqrt{{\text{log}}_{\rho }\left(1+\prod_{j=1}^{n}{\left({\rho }^{{{r}_{\alpha }^{2}}_{ij}}-1\right)}^{{\varphi }_{j}}\right)}\end{array}\right)$$

and

22 $${\beta }_{i}^{WPA}=Cr-PyFFW{G}_{min}\left({\alpha }_{1},{\alpha }_{2},\dots ,{\alpha }_{n}\right)=\left(\begin{array}{c}\sqrt{{\text{log}}_{\rho }\left(1+\prod_{j=1}^{n}{\left({\rho }^{{{\mu }_{\alpha }^{2}}_{ij}}-1\right)}^{{\varphi }_{j}}\right)},\\ \sqrt{1-{\text{log}}_{\rho }\left(1+\prod_{j=1}^{n}{\left({\rho }^{1-{{\nu }_{\alpha }^{2}}_{ij}}-1\right)}^{{\varphi }_{j}}\right)},\\ \sqrt{1-{\text{log}}_{\rho }\left(1+\prod_{j=1}^{n}{\left({\rho }^{1-{{r}_{\alpha }^{2}}_{ij}}-1\right)}^{{\varphi }_{j}}\right)}\end{array}\right)$$

**Point (d):** Examined the ranking of preferences by investigating score functions and employing the convex formula of the WASPAS method as follows:

23 $${\beta }_{i}= \tau {\beta }_{i}^{WSA}+\left(1-\tau \right){\beta }_{i}^{WPA}$$

where $$\tau \in \left[\text{0,1}\right].$$

**Point (e):** Maintained results of ${\beta }_{i}$ into descending order, and the highest value of the ${\beta }_{i}$ is the best optimal option from all considerations of preferences.

### Experimental case study

Prioritizing the elements that affect the quality of teacher preparation is crucial to improving student learning outcomes. The availability of ongoing professional development, the credentials and expertise of trainers, the integration of pedagogical and technological capabilities, and the depth and relevance of the training material are all important considerations. Training programs must also be in line with the demands of the modern curriculum and classroom conditions. Teachers who appropriately prioritize these factors are better able to meet the requirements of a wide range of students, use efficient teaching techniques, and adjust to changing academic standards. Additionally, training programs’ evaluation and feedback systems guarantee that teachers are always improving their abilities.

It is impossible to exaggerate the importance of excellent teacher preparation in education. Because they have a direct impact on student learning, engagement, and achievement, well-trained teachers are the cornerstone of an effective educational system. Good training programs help instructors become more competent, creative, and self-assured so they can create inclusive and engaging learning environments. Furthermore, by improving teachers’ capacity to include creativity and critical thinking into their lessons, this kind of training eventually equips pupils for problems they may face in the real world. Thus, enhancing the caliber of teacher preparation is a calculated move that will raise educational standards generally and foster long-term social progress.

Due to insufficient teacher preparation, the quality of education in many developing nations continues to fluctuate. The government has started numerous training initiatives; however the results frequently fall short of expectations. To investigate and rank the major elements influencing the calibre of teacher preparation in public schools. High-quality teacher preparation is essential for academic success. Nonetheless, the training process is influenced by a wide range of qualitative and quantitative parameters. Finding and ranking the most important elements that affect teacher preparation programs’ efficacy is a challenge for educational institutions. A national education board observed that classroom procedures and student learning outcomes remained substantially unaltered in spite of frequent teacher training sessions. Inconsistencies in training delivery, irrelevant information, and a lack of post-training support were found during an internal review. In this experimental case study, we considered five different training institutes. $\left\{{W}_{1}, {W}_{2},{W}_{3}, {W}_{4,}{W}_{5}\right\}$ and evaluate a suitable institute for teacher training to improve skills and expertise in his field. The evaluation process of the training institute is under consideration of the following criteria:

Research and Development Activities.

Infrastructure and Learning Resources.

Curriculum Relevance and Practical Teaching Experience.

Faculty Qualifications and Expertise.

Assessment and Evaluation Methods.

**Fig. 3:**
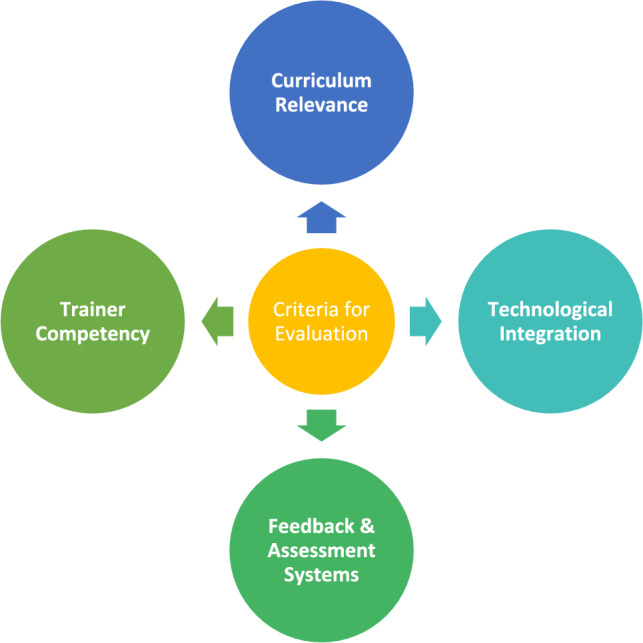
Criteria for the evaluation of specific training institutes.

The most important elements are trainer competency and continuous professional development, indicating that improving these areas can greatly raise the calibre of teacher preparation. In order to help educational policymakers make well-informed decisions, this method provides an organized and data-driven framework. Some characteristics of training institutes are also discussed in Fig. 3

### Decision-making process

This subsection designs the evaluation process of alternatives under different criteria and discusses decision-making models of the CRITIC-WASPAS method. To achieve the main goals of the manuscript, experts discussed their opinions under the system of Cr-PyF information. The assessment of training institutes using the stepwise decision algorithm of the CRITIC-WASPAS method is as follows.

#### Part 1

**Step 1:** We aim to establish expert judgments about five different training institutes based on five specific criteria in Tables 2, 3, 4.

**Table 2:** Judgment of the first expert listed in the decision matrix ${D}_{1}$.

	${\mathcal{U}}_{1}$			${\mathcal{U}}_{2}$			${\mathcal{U}}_{3}$			${\mathcal{U}}_{4}$			${\mathcal{U}}_{5}$		
${\mathbb{H}}_{1}$	$0.43$	$0.16$	$0.22$	$0.36$	$0.25$	$0.17$	$0.28$	$0.28$	$0.19$	$0.18$	$0.19$	$0.19$	$0.65$	$0.33$	$0.33$
${\mathbb{H}}_{2}$	$0.48$	$0.38$	$0.15$	$0.48$	$0.37$	$0.27$	$0.36$	$0.65$	$0.22$	$0.65$	$0.45$	$0.24$	$0.56$	$0.21$	$0.43$
${\mathbb{H}}_{3}$	$0.42$	$0.46$	$0.32$	$0.15$	$0.44$	$0.33$	$0.11$	$0.84$	$0.19$	$0.38$	$0.36$	$0.37$	$0.36$	$0.28$	$0.38$
${\mathbb{H}}_{4}$	$0.11$	$0.17$	$0.57$	$0.38$	$0.37$	$0.27$	$0.29$	$0.37$	$0.21$	$0.76$	$0.47$	$0.32$	$0.45$	$0.32$	$0.35$
${\mathbb{H}}_{5}$	$0.34$	$0.35$	$0.26$	$0.43$	$0.18$	$0.22$	$0.36$	$0.28$	$0.28$	$0.88$	$0.23$	$0.18$	$0.27$	$0.38$	$0.28$

**Table 3:** Judgment of the second expert listed in the decision matrix ${D}_{2}$.

	${\mathcal{U}}_{1}$			${\mathcal{U}}_{2}$			${\mathcal{U}}_{3}$			${\mathcal{U}}_{4}$			${\mathcal{U}}_{5}$		
${\mathbb{H}}_{1}$	$0.38$	$0.27$	$0.35$	$0.54$	$0.11$	$0.22$	$0.27$	$0.48$	$0.11$	$0.28$	$0.75$	$0.23$	$0.43$	$0.19$	$0.09$
${\mathbb{H}}_{2}$	$0.47$	$0.46$	$0.22$	$0.38$	$0.28$	$0.29$	$0.45$	$0.65$	$0.38$	$0.43$	$0.48$	$0.39$	$0.48$	$0.03$	$0.38$
${\mathbb{H}}_{3}$	$0.43$	$0.21$	$0.27$	$0.43$	$0.48$	$0.33$	$0.58$	$0.68$	$0.54$	$0.48$	$0.49$	$0.43$	$0.65$	$0.48$	$0.43$
${\mathbb{H}}_{4}$	$0.37$	$0.13$	$0.38$	$0.37$	$0.43$	$0.38$	$0.68$	$0.55$	$0.58$	$0.55$	$0.43$	$0.45$	$0.32$	$0.54$	$0.32$
${\mathbb{H}}_{5}$	$0.38$	$0.66$	$0.09$	$0.33$	$0.12$	$0.07$	$0.65$	$0.49$	$0.43$	$0.49$	$0.28$	$0.37$	$0.66$	$0.38$	$0.22$

**Table 4:** Judgment of the third expert listed in the decision matrix ${D}_{3}$.

	${\mathcal{U}}_{1}$			${\mathcal{U}}_{2}$			${\mathcal{U}}_{3}$			${\mathcal{U}}_{4}$			${\mathcal{U}}_{5}$		
${\mathbb{H}}_{1}$	$0.28$	$0.37$	$0.36$	$0.85$	$0.19$	$0.34$	$0.11$	$0.12$	$0.17$	$0.26$	$0.11$	$0.13$	$0.77$	$0.21$	$0.09$
${\mathbb{H}}_{2}$	$0.47$	$0.43$	$0.43$	$0.39$	$0.33$	$0.27$	$0.25$	$0.18$	$0.21$	$0.37$	$0.35$	$0.25$	$0.65$	$0.26$	$0.15$
${\mathbb{H}}_{3}$	$0.55$	$0.48$	$0.29$	$0.43$	$0.47$	$0.33$	$0.32$	$0.22$	$0.26$	$0.44$	$0.43$	$0.34$	$0.73$	$0.32$	$0.22$
${\mathbb{H}}_{4}$	$0.48$	$0.55$	$0.13$	$0.19$	$0.56$	$0.25$	$0.23$	$0.17$	$0.32$	$0.37$	$0.22$	$0.19$	$0.35$	$0.28$	$0.27$
${\mathbb{H}}_{5}$	$0.76$	$0.28$	$0.28$	$0.36$	$0.37$	$0.36$	$0.28$	$0.14$	$0.28$	$0.54$	$0.28$	$0.44$	$0.27$	$0.65$	$0.27$

**Step 2:** Computed the weight of experts $\left(0.3492, \text{0.3431,0.3077}\right)$ using Eq. 9 and the first three linguistic scales of Table 1.

**Step 3:** Aggregated expert’s judgments using proposed mathematical methodologies of Cr-PyFFWA operators and results depicted in Table 5.

**Table 5:** Aggregated decision matrix.

	${\mathcal{U}}_{1}$			${\mathcal{U}}_{2}$			${\mathcal{U}}_{3}$			${\mathcal{U}}_{4}$			${\mathcal{U}}_{5}$		
${\mathbb{H}}_{1}$	$0.3729$	$0.2484$	$0.3007$	$0.6466$	$0.1736$	$0.2303$	$0.2375$	$0.2615$	$0.1523$	$0.2432$	$0.2638$	$0.1806$	$0.6419$	$0.2379$	$0.1422$
${\mathbb{H}}_{2}$	$0.4735$	$0.4216$	$0.2376$	$0.4213$	$0.3248$	$0.2767$	$0.3678$	$0.4461$	$0.2621$	$0.5117$	$0.4263$	$0.2875$	$0.5675$	$0.1155$	$0.2997$
${\mathbb{H}}_{3}$	$0.4687$	$0.3581$	$0.2929$	$0.3614$	$0.4627$	$0.3300$	$0.4001$	$0.5307$	$0.3017$	$0.4355$	$0.4231$	$0.3797$	$0.6094$	$0.3519$	$0.3360$
${\mathbb{H}}_{4}$	$0.3541$	$0.2249$	$0.3185$	$0.3308$	$0.4435$	$0.2968$	$0.4724$	$0.3365$	$0.3415$	$0.6078$	$0.3625$	$0.3076$	$0.3800$	$0.3691$	$0.3135$
${\mathbb{H}}_{5}$	$0.5436$	$0.4098$	$0.1854$	$0.3772$	$0.1962$	$0.1737$	$0.4760$	$0.2760$	$0.3249$	$0.7085$	$0.2615$	$0.3048$	$0.4615$	$0.4505$	$0.2550$

#### Part 2

Here, we computed the degree of weights of criteria employing the stepwise procedure of the CRITIC method as follows:

**Step (a):** Determined score functions of each criterion associated with each alternative and estimated results shown in Table 6.

**Table 6:** Score functions matrix.

	$\mathcal{S}\left({\mathcal{U}}_{1}\right)$	$\mathcal{S}\left({\mathcal{U}}_{2}\right)$	$\mathcal{S}\left({\mathcal{U}}_{3}\right)$	$\mathcal{S}\left({\mathcal{U}}_{4}\right)$	$\mathcal{S}\left({\mathcal{U}}_{5}\right)$
${\mathbb{H}}_{1}$	$-0.1746$	0.0073	-0.1577	-0.1705	0.0310
${\mathbb{H}}_{2}$	$-0.1663$	-0.1686	-0.2310	-0.1685	-0.0585
${\mathbb{H}}_{3}$	$-0.1648$	-0.2651	-0.2744	-0.2343	-0.1017
${\mathbb{H}}_{4}$	$-0.1821$	-0.2555	-0.1723	-0.0967	-0.2137
${\mathbb{H}}_{5}$	$-0.1037$	-0.1102	-0.1465	0.0021	-0.1914
Max	$-0.1037$	0.0073	-0.1465	0.0021	0.0310
Min	$-0.1821$	-0.2651	-0.2744	-0.2343	-0.2137

**Step (b):** Table 7 presents the Cr-PyF orthonormal decision matrix obtained by Eq. 13.

**Table 7:** Orthonormal Cr-PyF decision-matrix.

	${\mathcal{U}}_{1}$	${\mathcal{U}}_{2}$	${\mathcal{U}}_{3}$	${\mathcal{U}}_{4}$	${\mathcal{U}}_{5}$
${\mathbb{H}}_{1}$	0.0953	1.0000	0.9122	0.2700	1.0000
${\mathbb{H}}_{2}$	0.2006	0.3544	0.3397	0.2785	0.6340
${\mathbb{H}}_{3}$	0.2208	0.0000	0.0000	0.0000	0.4576
${\mathbb{H}}_{4}$	0.0000	0.0354	0.7982	0.5822	0.0000
${\mathbb{H}}_{5}$	1.0000	0.5687	1.0000	1.0000	0.0911
Avg	0.3034	0.3917	0.6100	0.4261	0.4366
Stand	0.35720	0.369626	0.380524	0.341008	0.365189

**Step (c):** Table 7 also illustrates the results of the standard deviation by applying Eq. 14.

**Step (d):** Results of the correlation coefficient obtained from Eq. 15 and listed in Table 8.

**Table 8:** Correlation coefficient among criteria.

	${\mathcal{U}}_{1}$	${\mathcal{U}}_{2}$	${\mathcal{U}}_{3}$	${\mathcal{U}}_{4}$
1.0000	0.2136	0.3382	0.7146	-0.3794
0.2136	1.0000	0.6183	0.1958	0.6246
0.3382	0.6183	1.0000	0.7796	-0.1624
0.7146	0.1958	0.7796	1.0000	-0.6303
-0.3794	0.6246	-0.1624	-0.6303	1.0000
0.6741	0.9803	0.9794	0.7024	0.1653

**Step (e):** Find out the quantity of information associated with the criteria using Eq. 16 as follows:

$$\left(0.6741, 0.9803, 0.9794, 0.7024, 0.1653\right)$$

**Step (f):** Weights of criteria computed based on Eq. 17 as follows:

$$\left(0.1925, 0.2800, 0.2797, 0.2006, 0.0472\right)$$

#### Part 3

To find out the ranking of preferences under different key criteria, we applied the stepwise decision algorithm of the WASPAS method as follows.

**Step (a):** Since all key criteria are beneficial in the experimental case study employing Eq. 18, the results are shown as:

$$\left\{\begin{array}{c}\left(0.5436, 0.2249, 0.1854\right),\left(0.6466, 0.1736, 0.1737\right), \left(0.4760, 0.2615, 0.1523\right),\\ \left(0.7085, 0.2615, 0.1806\right), \left(0.6419, 0.1155, 0.1422\right)\end{array}\right\}$$

Table 9 of the decision matrix is obtained by employing Eq. 15.

**Table 9:** Normalied decision matrix.

	${\mathcal{U}}_{1}$			${\mathcal{U}}_{2}$			${\mathcal{U}}_{3}$			${\mathcal{U}}_{4}$			${\mathcal{U}}_{5}$		
${\mathbb{H}}_{1}$	$0.2415$	$0.2028$	$0.2536$	$0.3927$	$0.1479$	$0.1962$	$0.1609$	$0.2073$	$0.1322$	$0.1424$	$0.2091$	$0.1530$	$0.3909$	$0.2133$	$0.1245$
${\mathbb{H}}_{2}$	$0.3068$	$0.3442$	$0.2004$	$0.2559$	$0.2767$	$0.2358$	$0.2492$	$0.3536$	$0.2275$	$0.2995$	$0.3379$	$0.2435$	$0.3457$	$0.1035$	$0.2624$
${\mathbb{H}}_{3}$	$0.3037$	$0.2924$	$0.2471$	$0.2195$	$0.3942$	$0.2812$	$0.2711$	$0.4207$	$0.2618$	$0.2549$	$0.3354$	$0.3217$	$0.3711$	$0.3155$	$0.2942$
${\mathbb{H}}_{4}$	$0.2294$	$0.1836$	$0.2687$	$0.2009$	$0.3779$	$0.2529$	$0.3201$	$0.2667$	$0.2964$	$0.3557$	$0.2873$	$0.2605$	$0.2314$	$0.3309$	$0.2744$
${\mathbb{H}}_{5}$	$0.3522$	$0.3346$	$0.1564$	$0.2291$	$0.1672$	$0.1480$	$0.3225$	$0.2188$	$0.2820$	$0.4147$	$0.2073$	$0.2582$	$0.2811$	$0.4038$	$0.2232$

**Step (c):** Table 10 shows the results of WSA and WPA based on the proposed methodologies of Cr-PyFFWA and Cr-PyFFWG operators of Eqs. 21 and 22.

**Table 10:** Shows the results of the WSA and WPA models of the WASPAS method.

	WSA			WPA		
${\mathbb{H}}_{1}$	0.272503	0.188441	0.171971	0.228126	0.188441	0.183252
${\mathbb{H}}_{2}$	0.278496	0.307589	0.228887	0.27542	0.307589	0.230084
${\mathbb{H}}_{3}$	0.267397	0.363396	0.276863	0.261931	0.363396	0.279266
${\mathbb{H}}_{4}$	0.280101	0.28106	0.27014	0.265391	0.28106	0.271258
${\mathbb{H}}_{5}$	0.326505	0.224567	0.204454	0.311917	0.224567	0.221458

**Point (d):** Examined the ranking of preferences by investigating score functions and employing the convex formula of Eq. 23 and Table 11, which lists aggregated results.

**Table 11:** Results of alternatives or preferences.

	WSA	WPA	Results of alternatives
${\mathbb{H}}_{1}$	$-0.1419$	$-0.15822$	$-0.15088$
${\mathbb{H}}_{2}$	$-0.19459$	$-0.19593$	$-0.19532$
${\mathbb{H}}_{3}$	$-0.23491$	$-0.23725$	$-0.2362$
${\mathbb{H}}_{4}$	$-0.2024$	$-0.20683$	$-0.20484$
${\mathbb{H}}_{5}$	$-0.14776$	$-0.15959$	$-0.15427$

**Fig. 4:**
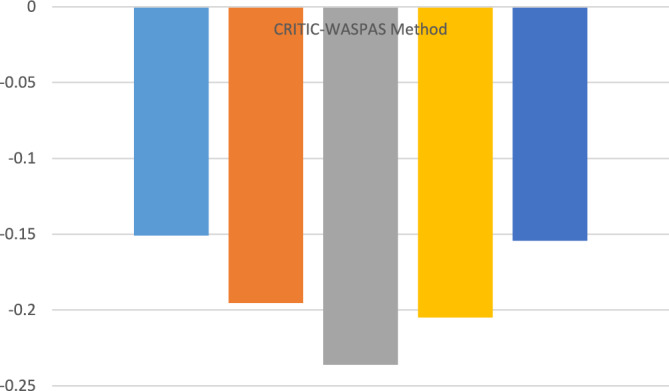
Ranking of preferences based on computed results by the CRITIC-WASPAS method.

**Point (e):** Maintained aggregated results of ${\mathbb{H}}_{i}$ in descending order and computed ranking of preferences as follows ${\mathbb{H}}_{1}\succ {\mathbb{H}}_{5}\succ {\mathbb{H}}_{2}\succ {\mathbb{H}}_{4}\succ {\mathbb{H}}_{3}$. Figure 4 also shows the graphical behavior of computed score functions based on the CRITIC-WASPAS method.

## Comparative study

A comparative study of existing mathematical approaches in decision-making highlights the strengths and limitations of traditional mathematical models. To achieve the goal of this section, we employed existing mathematical approaches and decision-making models on expert judgments. For this purpose, we considered some existing mathematical approaches of Hamacher aggregation operators based on the Cr-PyF framework^65^. Moreover, we also studied other decision-making models under the system of discussed terminologies of the fuzzy framework^62,66^.

We computed the ranking of preferences by employing the mathematical approaches of Hamacher aggregation operators ${\mathbb{H}}_{5}\succ {\mathbb{H}}_{2}\succ {\mathbb{H}}_{1}\succ {\mathbb{H}}_{4}\succ {\mathbb{H}}_{3}$. Furthermore, we also applied decision-making methodologies and aggregation operators of^62,66^. From^62^ and^66^, we acquired the ranking of alternatives ${\mathbb{H}}_{2}\succ {\mathbb{H}}_{5}\succ {\mathbb{H}}_{1}\succ {\mathbb{H}}_{3}\succ {\mathbb{H}}_{4}$ and ${\mathbb{H}}_{2}\succ {\mathbb{H}}_{1}\succ {\mathbb{H}}_{5}\succ {\mathbb{H}}_{4}\succ {\mathbb{H}}_{3}$ respectively. Nazir et al.^67^ explored the novel theory of circular pythagorean fuzzy situation with Hamy mean models. They also derived some mathematical models of Hamy mean operators to manage vague type information, and obtained a ranking of alternative ${\mathbb{H}}_{1}\succ {\mathbb{H}}_{5}\succ {\mathbb{H}}_{2}\succ {\mathbb{H}}_{4}\succ {\mathbb{H}}_{3}$ after applying existing Hamy mean operators. Bozyiǧit et al.^68^ initiated aggregation models of Choquet integral operators to resolve real-life applications and investigated the ranking of alternatives ${\mathbb{H}}_{2}\succ {\mathbb{H}}_{5}\succ {\mathbb{H}}_{1}\succ {\mathbb{H}}_{3}\succ {\mathbb{H}}_{4}$. We examined previous mathematical approaches and decision-making models, which are very simple. Sometimes, decision-makers face crucial challenges due to incomplete human judgment and redundant information about any object. However, some existing mathematical aggregation operators^69–71^ are also applied to consider expert’s judgments listed in Tables 2, 3, 4. We can observe that aggregation operators^69–71^ are unable to manage considered human opinion due to limited structure and key features.

In contrast, the CRITIC-WASPAS method offers a more robust and objective alternative. The CRITIC component calculates weights based on the variability and conflict among criteria, ensuring that the importance of each criterion is derived from data rather than subjective input. The WASPAS method then combines the strengths of the WSA and WPA, allowing for both additive and multiplicative performance evaluations, which enhances the reliability of ranking alternatives. Empirical comparisons and case studies have shown that CRITIC-WASPAS consistently yields more accurate and consistent results, particularly in complex decision-making environments. Its superior performance lies in minimizing human bias, capturing inter-criteria dynamics, and improving decision robustness, thereby proving its advantage over conventional MAGDM methods.

## Conclusion

This manuscript initiated a novel approach to the multi-attribute decision-making (MADM) problem to resolve complicated real-life applications having redundant and insufficient information about different preferences. To manage uncertainty in expert judgments and human opinions, establish robust decision-making methodologies and mathematical aggregation operators. We modified the notion of Cr-PyFSs with appropriate operational laws and comparison rules. A family of Frank aggregation operators is developed under the Cr-PyF framework, namely circular pythagorean fuzzy weighted average (Cr-PyFFWA) and circular pythagorean fuzzy weighted geometric (Cr-PyFFWG) operators. The derived mathematical models guaranteed both analytical robustness and practical application by combining the CRITIC-WASPAS method to evaluate the performance of different options. The CRITIC-WASPAS method plays a significant role in decision-making problems by providing a balanced and data-driven approach to evaluating and ranking alternatives. The CRITIC method is employed to determine the weight of each criterion by analyzing the variability and conflict among them. We modified a stepwise decision algorithm for resolving a group of expert’s opinions under Cr-PyF information and discussed the decision-making approach of the CRITIC-WASPAS method. To showcase the robustness of the CRITIC-WASPAS method, we discussed a numerical example to evaluate a suitable training institute under different key features and conflicting criteria. A comprehensive comparative study is also established to reveal the superiority and efficiency of the discussed approaches with existing mathematical terminologies.

The Cr-PyFS has a lot of advantages and key features that are used to handle uncertainty in human judgments. However, the discussed framework has various limitations that cannot be ignored during the aggregation process. The Cr-PyF framework is unable to handle human judgment when expert opinion has more than three components. To address such a situation, we can derive broader mathematical approaches under the system of complex picture fuzzy sets, bipolar fuzzy sets, and the complex t-spherical fuzzy framework.

## Data Availability

The datasets used and/or analysed during the current study available from the corresponding author on reasonable request.

## Abbreviations

Cr-PyFS: Circular pythagorean fuzzy set
Cr-PyFFWA: Circular pythagorean fuzzy frank weighted average
Cr- PyFFWG: Circular pythagorean fuzzy frank weighted geometric
IFS: Intuitionistic fuzzy set
FS: Fuzzy set
PyFS: Pythagorean fuzzy set
q-ROFS: Q-rung orthopair fuzzy set
AOs: Aggregation operators
Cr-PyFV: Circular pythagorean fuzzy value
CRITIC: Criteria importance through the intercriteria correlation
WASPAS: Weighted aggregated sum product assessment
Cr-IFS: Circular intuitionistic fuzzy set
MADM: Multi-attribute decision-making
IVIFS: Interval-valued IFS
MCDM: Multi-criteria decision-making
TOPSIS: Technique for Order of Preference by Similarity to Ideal Solution

## List of symbols

$B$: Non-empty set
$$\mu$$: Membership grade
$\nu$: Non-membership grade
$\gamma$: Element of a non-empty set
$$r$$: Hestancy degree
$$G$$: Alternative
$$H$$: Attributes
$$r$$: Radius
$${\mathbb{S}}$$: Score functions
$$A$$: Accuracy functions
$$\alpha$$: Cr-PyFV
$$\rho$$: Frank parametric
$\varphi$: Weight of criteria
$$\chi$$: Scalar multiple
